# Pax2-Islet1 Transgenic Mice Are Hyperactive and Have Altered Cerebellar Foliation

**DOI:** 10.1007/s12035-016-9716-6

**Published:** 2016-02-03

**Authors:** Romana Bohuslavova, Nicole Dodd, Iva Macova, Tetyana Chumak, Martin Horak, Josef Syka, Bernd Fritzsch, Gabriela Pavlinkova

**Affiliations:** 1 Institute of Biotechnology CAS, Prumyslova 595, Vestec, Prague-West District, 25242 Czech Republic; 2 Institute of Experimental Medicine CAS, Prague, Czech Republic; 3 Institute of Physiology CAS, Prague, Czech Republic; 4 Department of Biology, University of Iowa, Iowa City, IA USA

**Keywords:** Islet1 transcription factor, Vestibular system, Cerebellum, Foliation defects, Hyperactivity, GABA signaling, Transgenic mouse, Purkinje cells, Calcium homeostasis, Age-related deterioration of Purkinje cells, Attention deficit hyperactivity disorder

## Abstract

The programming of cell fate by transcription factors requires precise regulation of their time and level of expression. The LIM-homeodomain transcription factor Islet1 (Isl1) is involved in cell-fate specification of motor neurons, and it may play a similar role in the inner ear. In order to study its role in the regulation of vestibulo-motor development, we investigated a transgenic mouse expressing *Isl1* under the *Pax2* promoter control (*Tg* ^*+/−*^). The transgenic mice show altered level, time, and place of expression of Isl1 but are viable. However, *Tg* ^*+/−*^ mice exhibit hyperactivity, including circling behavior, and progressive age-related decline in hearing, which has been reported previously. Here, we describe the molecular and morphological changes in the cerebellum and vestibular system that may cause the hyperactivity of *Tg* ^*+/−*^ mice. The transgene altered the formation of folia in the cerebellum, the distribution of calretinin labeled unipolar brush cells, and reduced the size of the cerebellum, inferior colliculus, and saccule. Age-related progressive reduction of calbindin expression was detected in Purkinje cells in the transgenic cerebella. The hyperactivity of *Tg* ^*+/−*^ mice is reduced upon the administration of picrotoxin, a non-competitive channel blocker for the γ-aminobutyric acid (GABA) receptor chloride channels. This suggests that the overexpression of Isl1 significantly affects the functions of GABAergic neurons. We demonstrate that the overexpression of Isl1 affects the development and function of the cerebello-vestibular system, resulting in hyperactivity.

**Electronic supplementary material:**

The online version of this article (doi:10.1007/s12035-016-9716-6) contains supplementary material, which is available to authorized users.

## Introduction

The vestibular system of the ear provides a major input for balance [1]. Hair cells located within the five vestibular epithelia (the utricle, the saccule, and the lateral, superior, and posterior semicircular canal cristae) receive and convert stimuli in the three cardinal planes into electric signals [2]. The extracted information reaches the ipsilateral vestibular nucleus complex (VCN) in the brainstem and cerebellum [3, 4] via bipolar neurons, which form the vestibular part of the eighth cranial nerve. The region of the cerebellum that communicates most intimately with the vestibular system is the vestibulo-cerebellum, receiving afferents primarily from the vestibular ganglion and vestibular nuclei [4]. The cerebellum also receives proprioceptive input [5] and is part of a motor control loop to modify cortical signals for smooth, integrated movements [6] of the extraocular and skeletal muscles [7, 8]. The vestibular and proprioceptive signals are further processed and integrated together with other sensory, motor, and associative signals in the striatum, a central brain area for motor control (reviewed in [9]). The motor output pathways are regulated by the cerebellum and the striatum [9]. A connection between inner ear dysfunction, behavioral disorders such as hyperactivity and circling phenotype, and the striatum has recently been shown [10].

The insulin gene enhancer protein Islet1 (Isl1), a LIM-homeodomain transcription factor, contains two LIM domains which act as protein–protein interaction motifs and a homeodomain for recognizing and binding to specific DNA sequences, the primary structure of which is highly conserved among species. The combinations of LIM-homeodomain proteins form a transcriptional “LIM code” required for the specification and maintenance of different cell types during development [11, 12]. A LIM code is particularly well characterized for the developmental program of motor neurons [11, 13]. A LIM code defines the subtypes of motor neurons with the ability to select distinct axonal pathways, to recognize specific targets in the periphery, and to regulate viability. Isl1 is required for the differentiation and survival of motor neurons [14–16]. Isl1 is also essential for the development of striatonigral neurons [17, 18], and Isl1 expression in the ear suggests a role in cell lineage specification and differentiation of prosensory progenitors [16, 19, 20] possibly in interaction with other LIM-homeodomain factors [12]. The precise function of Isl1 in the development of the inner ear and the vestibular system-mediated motor coordination is unknown due to the early lethality of *Isl1* null mutants.

To further understand the function of Isl1, we used an overexpression model of *Isl1* under the *Pax2* regulatory sequence to explore the gain-of-function role of *Isl1* in the developing cerebellar and vestibular system. *Pax2* is one of the earliest genes to be expressed in the pre-otic region [21] and the midbrain/hindbrain region, giving rise to the cerebellum [22, 23]. Pax2 is a key regulator of otic cell identity and placode morphogenesis [24], and Pax2 combined with Pax8 is essential for mouse ear development with Pax2 playing a major role in cochlea development [25]. Pax2 is also involved in the specification of the midbrain/hindbrain region [26] including the formation of the cerebellum [23, 27]. *Pax2* expression at E7.5 initiates the partitioning of the midbrain/hindbrain region. Starting at E13.5, *Pax2* is expressed in prospective γ-aminobutyric acid (GABA) interneuron precursors in the cerebellar cortex, which sequentially generate different types of inhibitory interneurons according to an inside out progression: first are GABAergic neurons in the cerebellar nuclei, then Golgi and Lugaro cells in the granular layer, and finally basket and stellate cells in the molecular layer [28]. *Pax2* expression is downregulated when these interneurons mature and establish functional synaptic contacts with their targets [23].

Previously, we showed Isl1 to play a role in auditory system maintenance [29]. The transgenic expression of *Isl1* under *Pax2* regulatory sequences impaired the maintenance and function of hair cells of the organ of Corti with an early onset of age-related hearing loss, reflected in reduced otoacoustic emissions and the deterioration of the medial olivocochlear efferent system derived from facial motoneurons [30]. Additionally, the mutant mice exhibited increased levels of motor hyperactivity, including augmented locomotion and circling behavior, compared to *WT* littermates. In the current study, we present data showing that *Isl1* overexpression also causes some aberrant development of the vestibular system and the central nervous system, in particular the cerebellum, which may relate to hyperactivity.

## Materials and Methods

### Generation of Transgenic Mice

The use of animals in this study was conducted in accordance with the Guide for the Care and Use of Laboratory Animals (NIH Publication No. 85-23, revised 1996). All animal procedures were approved by the Animal Care and Use Committee of the Institute of Molecular Genetics, Academy of Sciences of the Czech Republic, and all efforts were made to minimize suffering. The experimental mice were housed in a controlled environment (23 °C, 12 h light/dark cycle) with free access to water and standard chow diet. All experiments were performed with both male and female littermate mice that were either wild-type or heterozygous *Pax2-Isl1* transgenic mice [Tg(*Pax2-Isl1*)Gp300] (*Tg* ^*+/−*^) on an FVB (*WT*) background (strain code 207, Charles River). *Tg* ^*+/−*^ mice were generated as described previously [29]. Genotyping was carried out from tail DNA by PCR using 5′ primer (located in *Pax2* regulatory element), 5′-AAG TTG AGT TTG AGA GGC GAC ACG-3′, and 3′ primer (located in *Isl1* gene), 5′-TTG GCG CAT TTG ATC CCG TAC AAC-3′ yielding a 400-bp amplicon. PCR was preformed over 35 cycles at 95 °C for 30 s, 63 °C for 30 s, and 72 °C for 30 s. The amplification products were run on agarose gels and visualized by ethidium bromide staining.

### Immunohistochemistry

Mice were perfused with 4 % paraformaldehyde (PFA), and temporal bones were dissected and fixed in 4 % PFA for 30 min. Sensory organs were dissected in phosphate-buffered saline (PBS) and decalcified in 0.12 M ethylendiaminotetraacetic acid. For brain dissections, the mice were first perfused with 4 % PFA and brains were stored overnight at 4 °C in 4 % PFA. The brains were sectioned in the sagittal plane at 80 μm/section using a vibratome and transferred free-floating into microplates containing 0.4 % PFA. The sections were defatted in 70 % ethanol for a minimum of 1 h and blocked with 2.5 % normal goat serum in PBS with 0.5 % Tween20 for 1 h. For histological analyses, dissected tissues were fixed with 4 % PFA in PBS (pH 7.4) at 4 °C overnight, dehydrated, and embedded in paraffin. Paraffin-embedded brains were cut in 7-μm sections, and tissue sections were stained with hematoxylin and eosin. The following dilutions of antiserum were used for immunohistochemistry: anti-Islet1 (no. 39.4D5, Developmental Hybridoma Bank, Iowa City, IA, USA) 1:200, anti-myosin 7a (Myo7a, no. 028918, Sigma-Aldrich) 1:500, anti-Pax2 (no. PRB-276P, Covance) 1:100, anti-acetylated tubulin (no. T6793, Sigma-Aldrich) 1:400, anti-calretinin (no. sc-50453, Santa Cruz Biotechnology) 1:100, anti-neurofilament 200 (NF200, no. N4142, Sigma-Aldrich) 1:200, and anti-calbindin (no. C9848, Sigma-Aldrich) 1:250. The vibratome sections and whole mount samples were incubated with primary Ab for 72 h at 4 °C. Following several washing steps with PBS, the corresponding secondary antibodies (Alexa dyes 1:400, Jackson ImmunoResearch Laboratories) were added and incubated overnight at 4 °C. The sections and whole mounts were counter-stained with Hoechst stain, mounted with antifade mounting medium, and viewed using Zeiss 510 DUO laser confocal (sections), confocal Leica SPE (whole mount, sections), or fluorescent stereomicroscope Leica MZFLIII (sections). Measurements of the whole cerebellar area and the percentage of calretinin staining quantification were performed using ImageJ software version 1.46r (National Institutes of Health, Bethesda, MD, USA). Three different sagittal sections were taken per sample, and percentage area and staining were taken between them. The quantification of saccular and utricular macula areas stained with anti-Myo7a antibody and number of Myo7a^+^ cells per 100 μm^2^ was done with ImageJ.

### Lipophilic Dye-Tracing

Heads of the pups were removed and fixed for a minimum of 24 h in 4 % PFA. NeuroVue® dye-coated filter microstrips were cut to appropriate size pieces using microscissors and inserted into the brainstem and saccule nerve tracts and incubated at 60 °C for 4 days [31]. A two-color tracing system using NeuroVue® Maroon and Orange, which have 647 and 538 nm excitation, respectively, was applied. Progression of dye diffusion was monitored using fluorescent dissection scopes. On completion of dye diffusion, whole mounts of the inner ear and brain stem were prepared using glycerol and coverslips as spacers [32]. Images were taken using Leica confocal laser scanning system, and the stack of images was collapsed into a single plane. Images were organized into plates using Corel Draw.

### Gene Expression Analysis by RT-qPCR

Total RNA was isolated from the cerebellum halves of 1-month-old mice using TRIzol® Reagent (Thermo Fisher Scientific Inc., Waltham, MA, USA). After removing genomic DNA by DNase I treatment (Thermo Fisher Scientific Inc., Waltham, MA, USA), RNA concentration and purity were determined using NanoDrop ND-1000 (Thermo Fisher Scientific Inc., Waltham, MA, USA). Isolated RNA (1 μg) was reverse transcribed into cDNA (RevertAid H Minus First Strand cDNA Synthesis Kit, Thermo Fisher Scientific Inc., Waltham, MA, USA). The obtained cDNA samples were diluted 20×. Each reaction for qPCR analysis contained 4 μl diluted cDNA, 5 μl SYBR Green JumpStart Taq ReadyMix for qPCR (Sigma-Aldrich, St. Louis, MO, USA), 0.5 μl ultrapure water, 0.25 μl 10 mM forward primer, and 0.25 μl 10 mM reverse primer. The primer sequences are listed in Table S1. qPCR was performed with the initial activation at 94 °C for 120 s, followed by 39 cycles at 94 °C for 15 s, 60 °C for 30 s, and 72 °C for 30 s using the CFX384™ Real-Time PCR Detection System (Bio-Rad Laboratories, Hercules, CA, USA). The –ΔΔCq method was used to quantify the relative mRNA expression [33] with *Hprt1* as a reference gene [34]. The *Isl1* reaction products were analyzed using agarose gel electrophoresis. Equivalent aliquots of each amplification reaction were separated on a 2 % agarose gel containing 0.5 μg/ml ethidium bromide.

### Behavior and Systemic Drug Testing

All testing was carried out during the light cycle. We only used 7–13-week-old males for all behavior tests. The mice were individually placed in a chamber (37 cm length × 20 cm width × 14.5 cm height) and allowed to acclimatize for 30 min before testing. To analyze locomotor activity in an open-field environment, the mice were recorded (Sony DCR-SX85Camcoder) in 9-s sequences during a 20-min period. Six movement sequences were analyzed per mouse. The average of the total distance traveled over a 2-min time period and the average velocity were quantified using the NIH ImageJ program with the Manual Tracking Plug-in (http://imagej.nih.gov/ij/download.html). The vestibular function was evaluated by the ability of the mice to right themselves in the air (air-righting reflex) when held supine and dropped onto a soft surface from a height of 50 cm [35]. The average percentage of trials of each mouse landing on all four feet from five attempts/mouse was determined.

Rotarod assays were performed using the rotarod apparatus (Rota Rod 47600, Ugo Basile) to assess fine motor coordination and balance [36, 37]. Briefly, during the acclimatization period, mice with their heads in the direction of rotation were loaded on the rotarod at an initial speed of 4 rpm. This speed was maintained for 2 min and, if mice fell during this period, they were placed back on the rotarod. For the experimental measurements, the drum was slowly accelerated to a speed of 4–40 rpm for a maximum of 300 s for each trial. The latency to fall off the rotarod within 300 s was recorded. If the mouse clung to the grip of the rotating drum and failed to resume normal performance for three consecutive revolutions, the sensor was manually triggered. Mice were tested in three consecutive trials in one session per day with a 15-min rest period between each trial.

The baseline levels of open-field measurements for each mouse were compared the day before and after drug administration at indicated times. The drugs were injected intraperitoneally, and open-field activity was recorded after injection at indicated times. We used the dopaminergic antagonist haloperidol and a long-acting haloperidol decanoate (0.25 mg/kg, [10, 38]), the glutamatergic *N-*methyl-d-aspartate receptor antagonist ketamine (3 mg/kg, [39]), picrotoxin, a non-competitive channel blocker for the GABA receptor chloride channels (1 mg/kg, [40]), and α-lobeline, nicotine acetylcholine receptor antagonist (1 mg/kg [41]) at a volume of 10 μl/1 g of mouse weight in sterile buffered saline or sesame oil for haloperidol decanoate.

### Auditory Brainstem Response Testing

For auditory brainstem response (ABR) recording, needle electrodes were placed subcutaneously on the vertex (active electrode) and in the neck muscles (ground and reference electrodes). The click-evoked responses were recorded (angular pulse with alternating polarity, duration 0.1 ms, repetition rate of 11 Hz). Acoustic stimuli were conveyed to the animal in free-field conditions via a two-way loudspeaker system (Jamo® woofer [Denmark] and SEAS® T25CF 002 tweeter [Norway]) placed 70 cm in front of the animal’s head. The signal was processed with a TDT System III Pentusa Base Station and analyzed using BioSig™ (TX, USA) software. The ABR responses of five *WT* and five *Tg* ^*+/−*^ mice were recorded.

### Statistical Analysis

The differences between *WT* and *Tg* ^*+/−*^ in behavior tests were tested using one-way ANOVA with Bonferroni’s multiple comparison test and two-way repeated measures ANOVA; qPCR expression, Myo7, and calretinin data were analyzed by Student’s *t* test (significance assigned at the *P* < 0.05 level; GraphPad, 2005; San Diego, USA).

## Results

### Behavioral Changes in *Tg*^*+/−*^ Mice

**Fig. 1:**
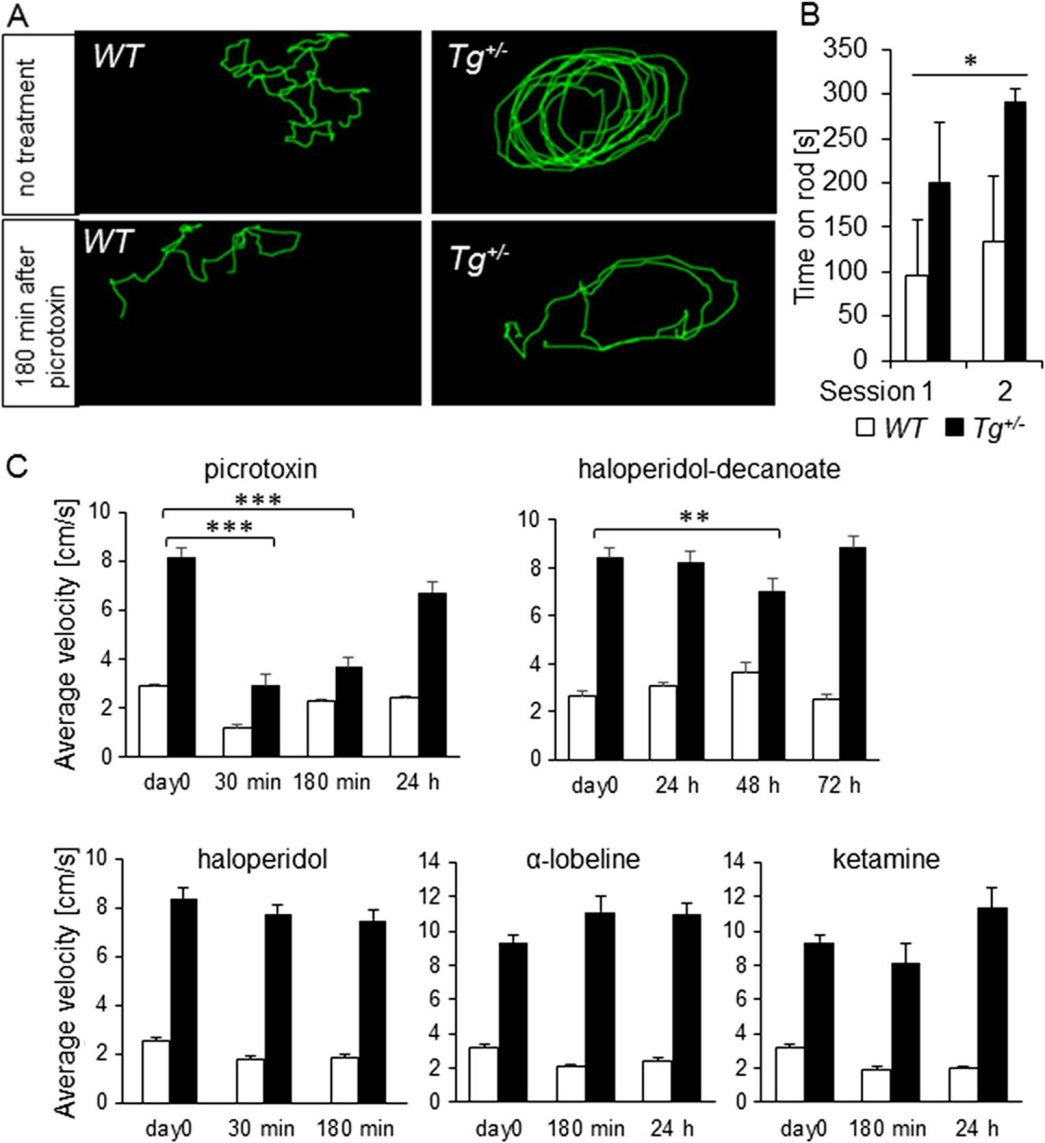
Behavior tests in an open field. *Tg* ^*+/−*^ mice display GABA receptor-mediated increased locomotor activity. **a** Traces of locomotion in an open field show significant hyperactivity and circling of *Tg* ^*+/−*^ mice. **b** Motor coordination of *WT* (*n* = 6) and *Tg* ^*+/−*^ (*n* = 3) mice on the accelerating rotarod was analyzed in three trials/session (repeated measures ANOVA: genotype effect, ***P < 0.0001; session effect, *P < 0.0117). The values represent means of three trials/session ± SEM. **c** Quantification of mouse locomotion in an open field showing that picrotoxin significantly reduced hyperactivity of mutants (*n* = 6 *Tg* ^*+/−*^, *n* = 5 *WT*) but did not correct circling. There was a significant alleviation of the locomotor activity of *Tg* ^*+/−*^ mice compared to *WT* after the application of haloperidol-decanoate at 48 h. Data represent mean ± SEM (**P < 0.01; ***P < 0.001)

In all experiments, only heterozygous *Pax2-Isl1* transgenic mice (*Tg* ^*+/−*^) were analyzed. Homozygosity for the [Tg(Pax2-Isl1)] allele is associated with severe abnormalities in the mid-hindbrain region and signs of developmental arrest at E10.5 [29]. Although the heterozygous transgenic mice are viable, approximately 40 % of the *Tg* ^*+/−*^ pups do not survive the first 2 days of life, suggesting altered early postnatal development [29]. The surviving adult *Tg* ^*+/−*^ mice exhibited significant (*P* < 0.001) increased levels of motor activity and circling behavior compared to *WT* littermates, suggesting defects in the vestibular system (Fig. 1a; supplemental files: movie M1 and M2). During open-field observations, the mutant mice did not display any rapid sideway wagging movements of the head, rapid vertical bobbing movements of the head, or any sustained tonic contractions or tremor of the limbs or trunk. Hyperactivity and the abnormal circling behavior of *Tg* ^*+/−*^ mice started with full maturity (approximately at 6 weeks of age) and intensified with increasing age. The mice consistently displayed a unidirectional circling preference of either left-circling or right-circling. The average movement velocity of *Tg* ^*+/−*^ mice was significantly higher (8.5 ± 0.3 cm/s; *n* = 6) compared to *WT* (2.1 ± 0.1 cm/s; *n* = 6, *P* < 0.0001; Fig. 1a). The hyperactivity phenotype was associated with a lower body weight of *Tg* ^*+/−*^ mice (23.1 ± 0.49 g, *N* = 6, 7 weeks) compared to *WT* (28.5 ± 0.70 g, *N* = 5, *P* < 0.0001, *t* test) despite free access to the same food. A basic test of vestibular function, the air-righting test, showed both *Tg* ^*+/−*^ and *WT* mice landed on their feet most of the time dropped supine out of 50 cm height onto a soft padding. An additional test to measure motor functions was performed on the rotarod (Fig. 1b). The performance of *Tg* ^*+/−*^ mice in the accelerating rotarod motor learning paradigm was superior to *WT* littermates. Repeated measures ANOVA showed a significant genotype effect (*P* < 0.0001) and a significant session (time) effect (*P* < 0.0117). Unexpectedly, the performance of *Tg* ^*+/−*^ mice improved with the training as in the second session of the motor learning *Tg* ^*+/−*^ mice reached the maximum testing time of 300 s in all trials with one exception of a shorter trial period of 220 s. Both tests assessing motor coordination and balance showed that *Tg* ^*+/−*^ mice were hyperactive without any demonstrable motor deficiencies. An increase in locomotor activity is therefore not necessarily related to a dysfunction of the inner ear but rather implies an alternation of brain functions [10]. Consistent with this concept that the abnormal locomotor phenotype may originate in the brain instead of the ear, picrotoxin, a non-competitive channel blocker for the GABA receptor chloride channels normalized the open-field hyperactive behavior of *Tg* ^*+/−*^ mice. After 30 and 180 min, the hyperactivity of *Tg* ^*+/−*^ mice was decreased by 61 and 55 %, respectively, compared to untreated *Tg* ^*+/−*^ (*P* < 0.001; Fig. 1a, c; supplemental files: movie M1–4). The same dose of pictrotoxin did not significantly affect the locomotor activity in control littermates compared to untreated *control* mice. We tested the effects of glutamatergic *N*-methyl-d-aspartate receptor antagonist (ketamine), dopaminergic antagonist (haloperidol), and nicotine acetylcholine receptor antagonist (α-lobeline; Fig. 1c). In all behavioral tests, the difference in the average velocity between *Tg* ^*+/−*^ and *control* mice was significant in all treatment groups. However, treatment with ketamine and α-lobeline had no effect on the hyperactivity of *Tg* ^*+/−*^ mice. Haloperidol, which acts in the brain to alleviate hyperactivity in humans, did not have a significant effect, although a moderate decrease of locomotor activity was noticeable (Fig. 1c). For a long-acting effect, we used haloperidol-decanoate that attenuated the hyperactivity in *Tg* ^*+/−*^ by 23 % compared to untreated *Tg* ^*+/−*^ at 48 h after the application (*P* < 0.01; Fig. 1c). The same dose of haloperidol did not significantly affect *WT* littermates at any time points. The systemic responsiveness to picrotoxin and to haloperidol indicates a disruption of brain functions that regulate movement in addition to the vestibular system dysfunction of *Tg* ^*+/−*^ mice. The apparent effects of picrotoxin, a non-competitive channel blocker of the GABA receptor chloride channels, suggest that the hyperactivity of transgenic mice is associated with altered GABA signaling. Although a trend of decreased activity after haloperidol treatment is evident, in comparison to picrotoxin, the effect of haloperidol on locomotor activity of *Tg* ^*+/−*^ mice is less prominent.

### Changes in the Vestibular End Organs of *Tg*^*+/−*^ Mice

**Fig. 2:**
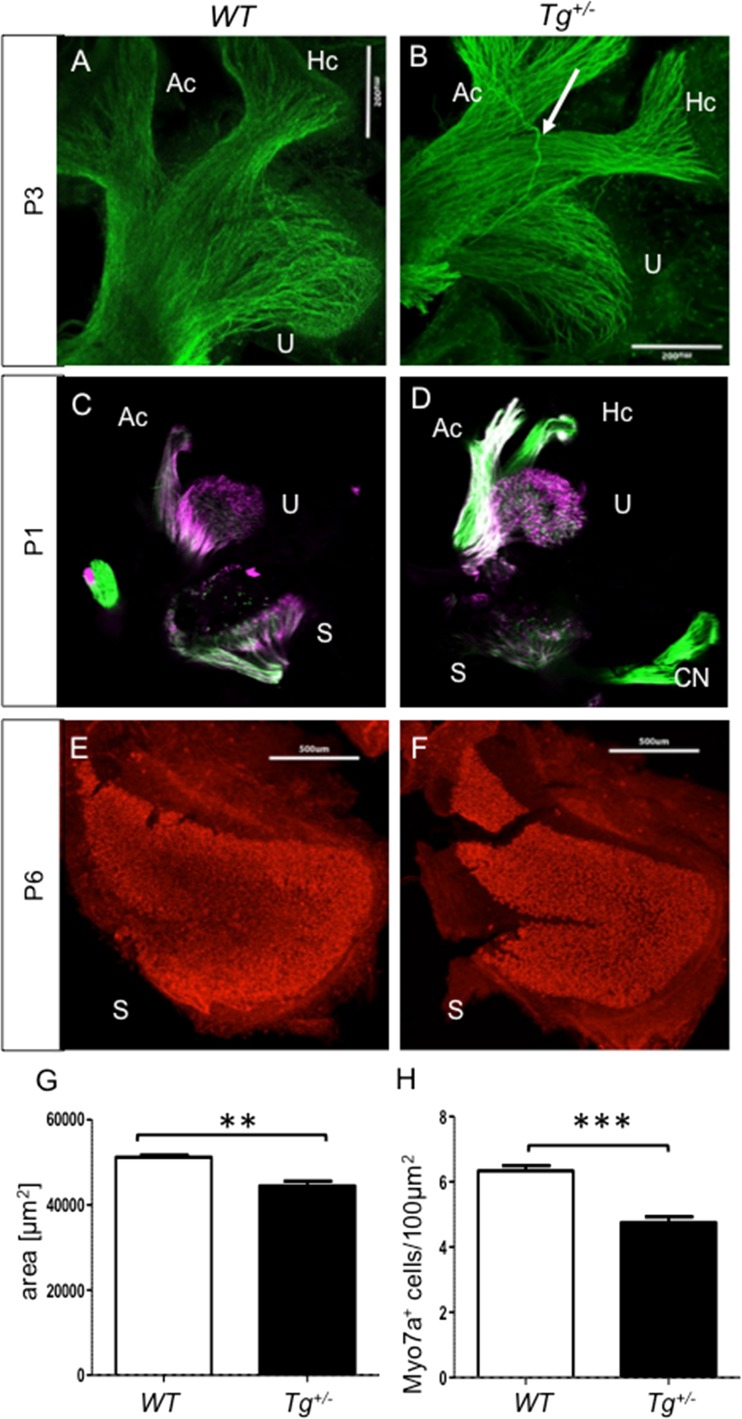
The pattern of innervation in the vestibular system at P3 (**a**, **b**). Similar dense innervation of *WT* and *Tg* ^*+/−*^ sensory epithelia is shown by anti-tubulin staining of the fibers in whole mount. A misguided nerve fiber with the same aberrant trajectory was repeatedly observed in *Tg* ^*+/−*^ (*white arrow*). *Scale bar* 500 μm. **c**, **d** Less fibers in the transgenic saccule at P1. The utricle and anterior and horizontal canals are typically labeled at the same intensity. Lipophilic dyes were injected into the cerebellum. **e**, **f** A reduction of sensory epithelium of the saccular maculae in *Tg* ^*+/−*^ (**e**) compared to *WT* (**f**) at P6. Hair cells are visualized using anti-Myo7a (*red*) in whole-mount immunohistochemistry. Quantification of area saccule (**g**) and counting of Myo7a^+^ cells per 100 μm^2^ (**h**) is done by ImageJ. The values represent means ± SEM (*N* = 3 individuals/group and 6 × 100 μm^2^/3 individuals/group). **P < 0.01; ***P < 0.001. *Scale bar* 500 μm. *U*, Utricle; *Ac*, anterior canal crista; *Hc*, horizontal canal crista; *S*, saccule

To determine whether the behavioral disorders of *Tg* ^*+/−*^ could be associated with inner ear changes, we analyzed the vestibular end organs and patterns of innervation. The saccule is the first sensory epithelium to differentiate and connect to the brainstem and cerebellum [3, 42]. We observed more branching in the saccule, more fibers going to the posterior canal, and more fibers already extending into the cerebellum earlier in the transgenic embryos compared to *WT* at E12.5 [29]. The overall patterns of vestibular innervation were further investigated in *Tg* ^*+/−*^ mice after birth by immunohistochemistry using an anti-acetylated tubulin antibody (Fig. 2a, b). The innervation of the anterior vertical canal crista (AC), horizontal canal crista (HC), and utricle (U) in *Tg* ^*+/−*^ pups was comparable with that in the wild type with the exception of occasional nerve fibers with an aberrant trajectory in *Tg* ^*+/−*^ (Fig. 2b, arrow). In addition, we used lipophilic dye-tracing to examine the innervation patterns of *WT* and mutant inner ears. At P1, the utricle and anterior and horizontal canals were labeled at the same fiber density in the *Tg* ^*+/−*^ and *WT* littermates (Fig. 2c, d). However, the labeling intensity of the saccule in the *Tg* ^*+/−*^ was much lower (Fig. 2d). To check if this staining difference is related to aberration in the sensory epithelium, we analyzed the size of the saccule and utricle in a whole mount preparation at P6. The size of the utricular maculae showed no measurable difference (data not shown), but the size of *Tg* ^*+/−*^ saccular maculae was significantly smaller (*P* < 0.01) compared to *WT* (Fig. 2e–g). Furthermore, the number of hair cells per 100 μm^2^ of the sensory epithelium of the saccule was significantly reduced (*P* < 0.001) in *Tg* ^*+/−*^ (Fig. 2h), seemingly corresponding to the reduced innervation (Fig. 2d).

**Fig. 3:**
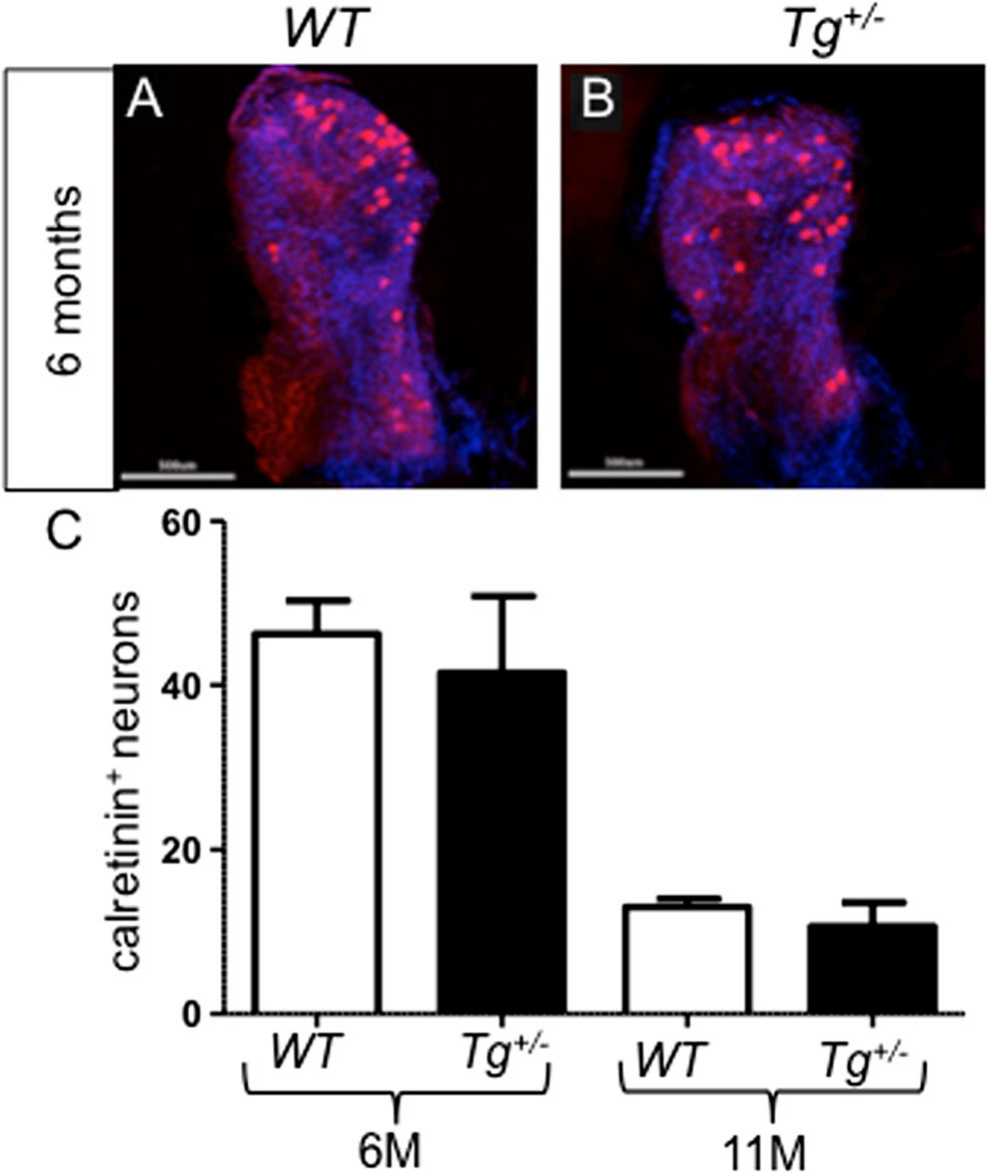
Total number of calretinin-labeled neurons in the vestibular ganglion of *WT* (**a**) and *Tg* ^*+/−*^ (**b**). **c** The number of calretinin^+^ neurons in *WT* and *Tg* ^*+/−*^ ganglia is similar at 6 months of age (*6M*) and it is declining with age at a similar rate in both *WT* and *Tg* ^*+/−*^ (11 months of age, *11M*). Single immunostaining with anti-calretinin (*red*) and visualization of nuclei with Hoechst (*blue*). *Scale bar* 500 μm

To further examine the properties of the vestibular afferents in the vestibular ganglion, we used calretinin, a marker of a selective population of large ganglion neurons that project centrally into the brainstem vestibular nuclei and the vestibular cerebellum [43–45]. The total number of calretinin^+^ neurons in the vestibular ganglion was not significantly altered in *Tg* ^*+/−*^ compared to *WT* littermates (Fig. 3a–c). Both *Tg* ^*+/−*^ and *WT* ganglia were similarly affected by the aging process and showed the well-known age-related decline [46] when we compared 6- and 11-month-old mice (Fig. 3c).

### Transgenic Isl1 Expression in the Cerebellum Causes Foliation and Cellular Changes

**Fig. 4:**
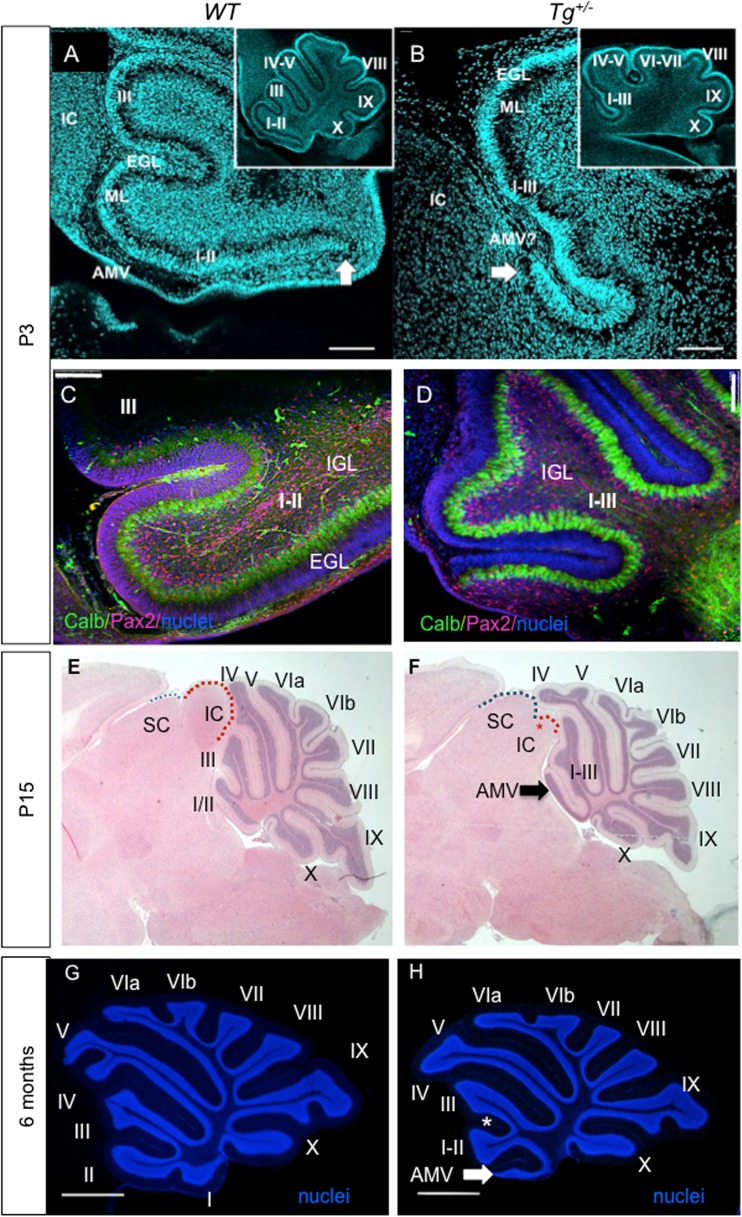
Changes in the cerebellum. **a**, **b** P3 sagittal sections using Hoechst nuclear staining show the different organizations in the control (*WT*) and mutant (*Tg* ^*+/−*^) littermate cerebellar foliation (insert **a**, **b**) and disorganization of lobule I + II. Note absence of a recognizable anterior medullary velum (*AMV*) and the rostral expansion of a hemilobe only in the transgenic mouse (*arrowhead*). **c**, **d** Pax2 (*red*) and calbindin (*green*; Purkinje cells) staining of sagittal sections of the anterior lobe of the cerebellar vermis at P3 shows a comparable distribution of Purkinje cells and Pax2^+^ cells in *WT* (C) and *Tg* ^*+/−*^ lobules (**d**). The altered foliation of lobules I–III is obvious in the *Tg* ^*+/−*^ cerebellum. **e**, **f** Hematoxylin-eosin staining of the brain sections at the level of vermis at P15. The predominant phenotype of altered formation of vermis lobules leading to the fusion of I–III and a hemilobule on top of or as part of the anterior medullary velum (*arrow*) is detected in the *Tg* ^*+/−*^ cerebellum. The remnant of the inferior colliculus (*IC*) is denoted by a *red asterisk* in the *Tg* ^*+/−*^ midbrain. The superior colliculus (*SC*) and IC are outlined by *blue*- and *red-dashed lines*, respectively. **g**, **h** The adult *Tg* ^*+/−*^ cerebellum shows the defect in the foliation of the anterior lobe compared to *WT* littermates as shown by Hoechst staining of the granule cell layer nuclei. The fissure (*) between anterior folia I/II and III failed to form properly, leading to the fusion of the lobules. A hemilobule is on top of or as part of the anterior medullary velum (*arrow*). The lobules IV–V in *Tg* ^*+/−*^ differ from controls. Roman numerals depict cerebellum lobules. *AMV*, anterior medullary velum; *Calb*, calbindin; *EGL*, external granule layer; *IGL*, internal granule layer; *IC*, inferior colliculus; *ML*, molecular layer; *SC*, superior colliculus. *Scale bar* 100 μm (**a**–**d**) and 1000 μm (**e**–**h**)

**Fig. 5:**
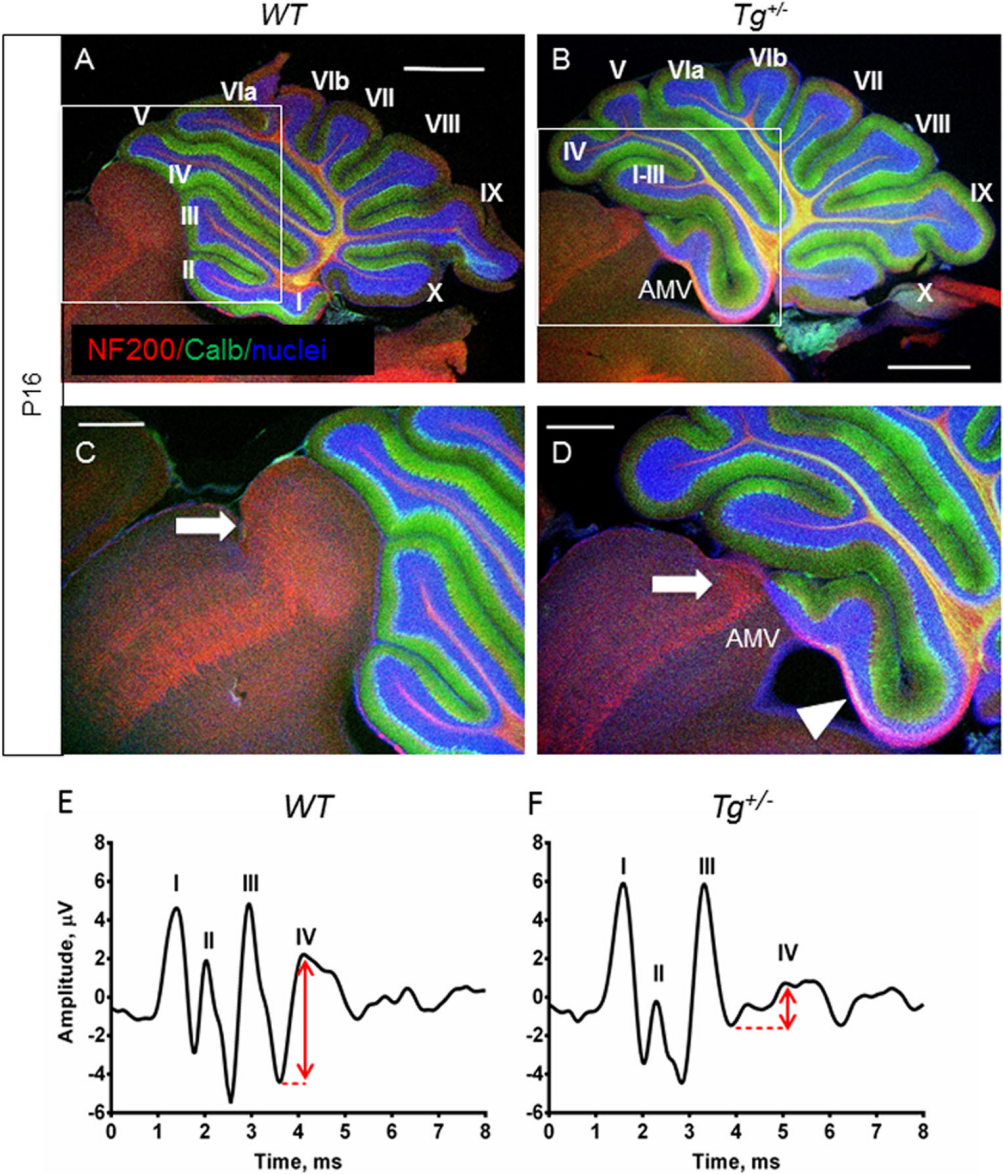
Changes in the inferior colliculus of transgenic mice. Representative confocal images shows the expression of NF200 (*red*) and calbindin (*green*) in cerebellar sections from P16 *WT* (**a**, **c**) and transgenic (**b**, **d**) mice. **c**, **d** The brachium of the inferior colliculus (*arrow*) is profoundly reduced in *Tg* ^*+/−*^ compared to *WT* mice. An *arrowhead* indicates an aberrant tract of white matter fibers in the transgenic cerebellum expanding along the hemilobe that is fused with the anterior medullary velum (*AMV*). **e**, **f** The auditory brainstem response (*ABR*) waveforms of 3-week-old mice to a click stimulus. Individual responses at 80 dB SPL click are represented. Major waves I–IV are indicated above the peaks. The results show that the amplitude of ABR wave IV is lower, and the latency of ABR waves is prolonged in *Tg* ^*+/−*^ compared to *WT. Scale bar* 1000 μm (**a**, **b**) and 500 μm (**c**, **d**)

Since the relatively minor changes found in the inner ear could not be matched to the obvious motor deficits of transgenic mice, we next analyzed the cerebellum, a motor control system [6]. The area of the cerebellum was compared using three near midsagittal sections through the cerebellar vermis. The *Tg* ^*+/−*^ cerebella were smaller (*P* < 0.0278) compared to control littermates at P8 (9.227 ± 0.6 mm^2^, *N* = 6 versus11.16 ± 0.3 mm^2^, *N* = 5). Changes differed by lobule. For example, lobule X and lobule IX trended to be smaller, but the difference was not significant (*P* > 0.05, *t* test). Transgenic mice had consistent foliation defects in the anterior lobe (I–V lobules) of the cerebellar vermis (Fig. 4). The predominant phenotype was the fusion of vermis lobules I–II and III. The fissure between anterior-folia I/II and III either failed to form, leading to the fusion of the lobules, or was shallower in the transgenic cerebella than in *WT* (Fig. 4d, f, h). Importantly, most of the transgenic mice had a hemilobule on top of or as part of the anterior medullary velum (Fig. 4b, f, h, arrow). A mild foliation defect in lobules IV and V was consistently detected in *Tg* ^*+/−*^ (Fig. 4g, h). In one of the 20 adult mutants analyzed, lobules VI–VIII failed to form. Additionally, sagittal sections of the *Tg* ^*+/−*^ brain revealed that the inferior colliculus was smaller (Fig. 4f). Although the brains of all adult *Tg* ^*+/−*^ mice analyzed (*n* = 20) appeared grossly normal, the inferior colliculus was noticeably reduced in the dorsal view of the adult brains (supplemental file: Fig. S1). The fiber bundle of the inferior colliculus reaching to the medial geniculate body (brachium of the inferior colliculus; BIC) was significantly reduced in the *Tg* ^*+/−*^ inferior colliculus, as shown by NF200 staining (Fig. 5c, d, arrow). Additionally, white matter fibers formed a distinctive tract in the *Tg* ^*+/−*^ cerebellum with an aberrant fiber bundle forming the outer layer of the anterior cerebellum (Fig. 5d, arrowhead). To analyze inferior colliculus activation, we performed ABR recordings. The amplitude of the IV wave was lower, and the latency of all ABR waves (I–IV) was prolonged in *Tg* ^*+/−*^ compared to *WT* mice (Fig. 5e, f). Since wave IV represents lateral lemniscus and inferior colliculus activation [47], the ABR data confirm functional abnormalities of the inferior colliculus of *Tg* ^*+/−*^ mice.

**Fig. 6:**
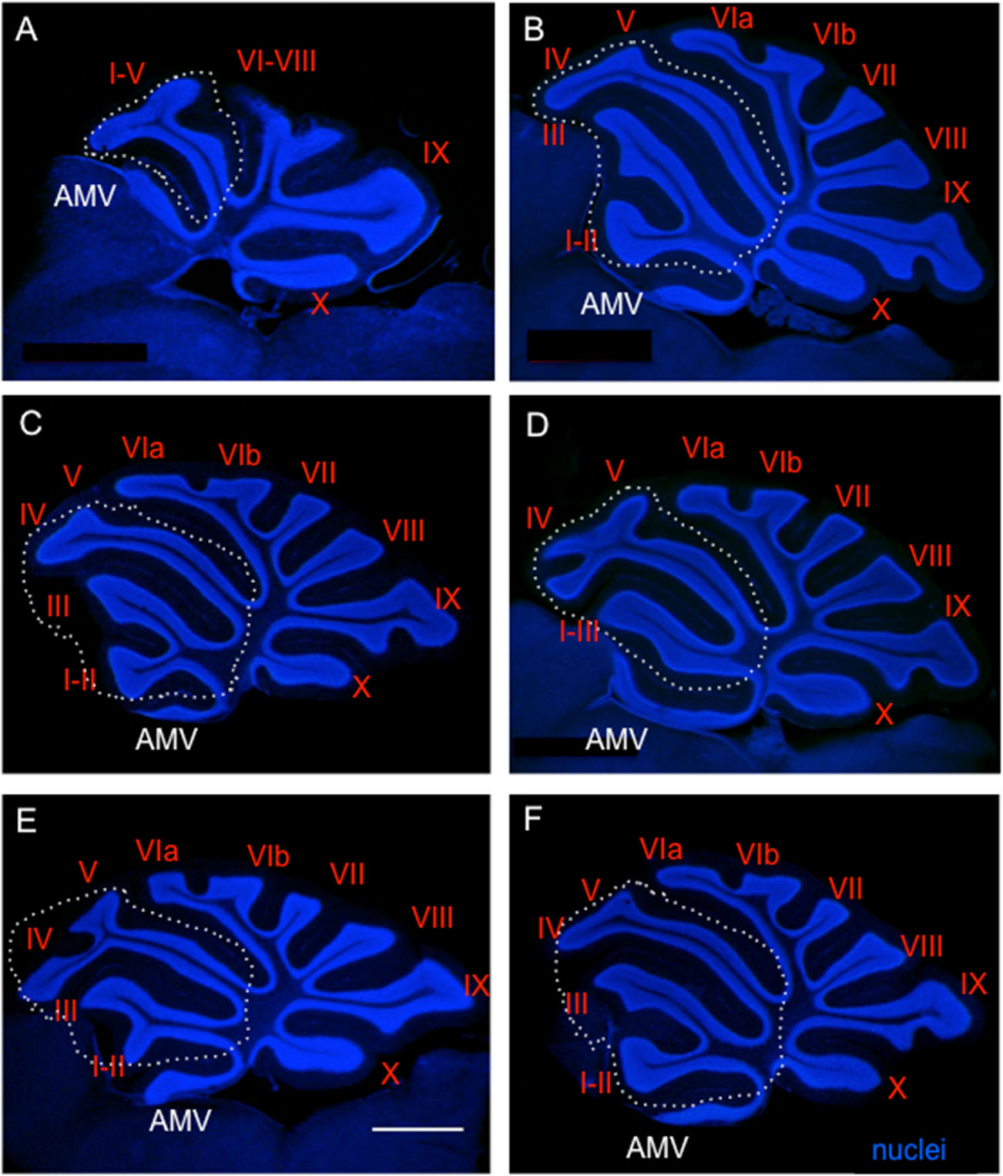
Morphological changes in the adult transgenic cerebellum. Hoechst staining of the granule cell layer nuclei of the cerebellum shows a differential penetrance leading to variable foliation defects in *Tg* ^*+/−*^. Severe foliation defects (**a**) compared to less affected *Tg* ^*+/−*^ (**b**–**f**). The formation of the anterior lobe (lobules I–V) is altered in all *Tg* ^*+/−*^. The area of the anterior lobe is outlined by *white dashed line* and shows defects in all transgenic mice, including the AMV aberration. *AMV*, anterior medullary velum. *Scale bar* 1000 μm

We observed a different penetrance of the cerebellar phenotype, from severe foliation defects with a significant reduction/fusion of lobules (Fig. 6a) to milder changes in the formation of lobules in the anterior lobe (Fig. 6b–f). One likely possibility for the variable phenotype is the mosaic expression of the transgene due to the heterozygosity of the mutation.

**Fig. 7:**
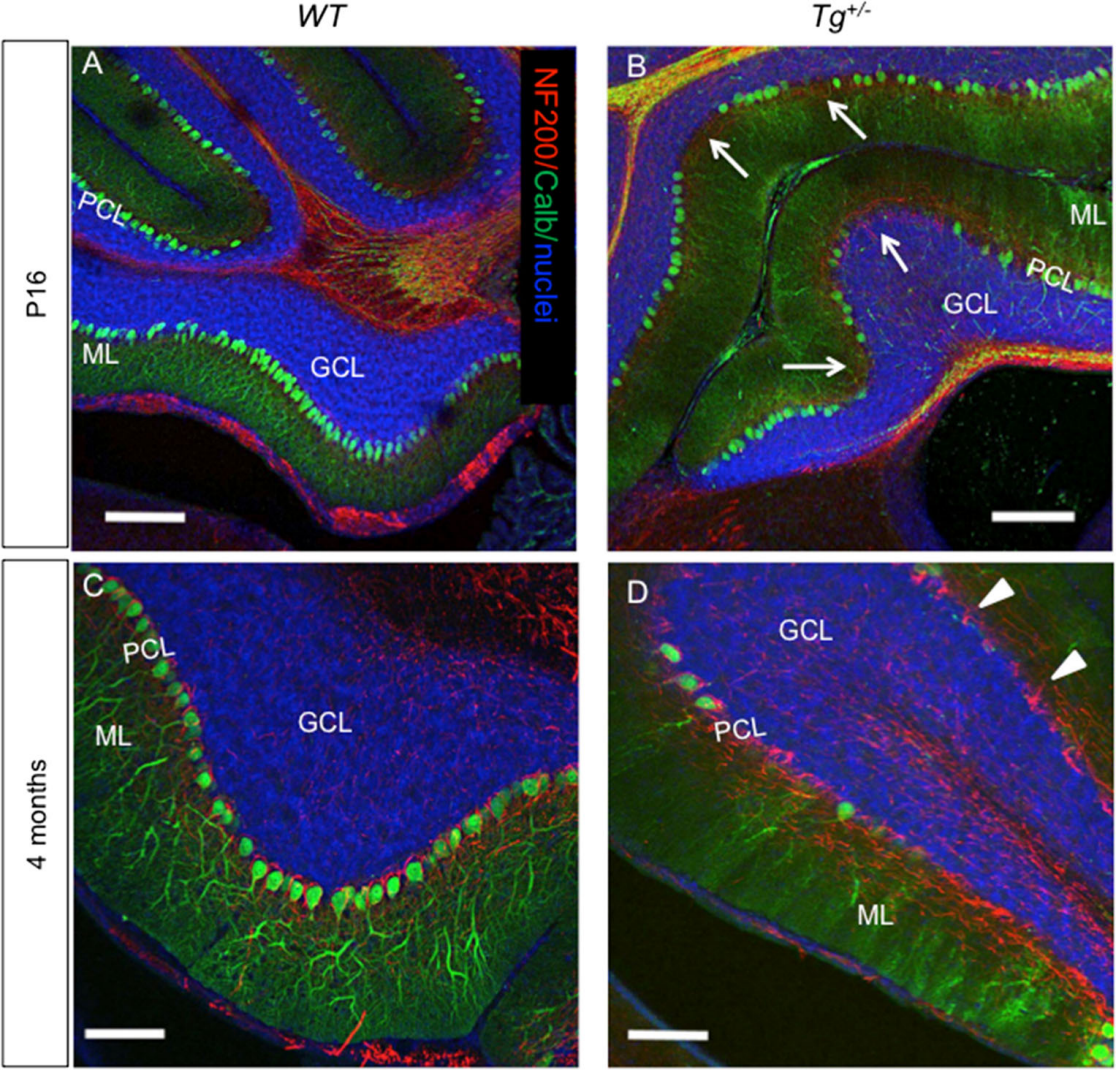
Changes in Purkinje cells in the anterior lobe (detail of lobules I–II). **a**, **b** Purkinje cells (*PCs*) are oriented in a monolayer with dendrites projecting into the ML at P16, as visualized by calbindin staining (*green*; *nuclear staining*, *blue*). More calbindin-negative PCs are visible in the *Tg* ^*+/−*^ anterior lobe (**b**, *arrows*). The density of PC dendrites stained by calbindin is noticeably reduced in *Tg* ^*+/−*^ compared to *WT* (**a**) at P16. **c**, **d** A profound reduction of calbindin expression in PCs and PC dendrites in the ML progresses with increasing age in the *Tg* ^*+/−*^ anterior lobe (**d**), as visualized by lack of staining with anti-calbindin. Anti-NF200 staining (*red*) of basket interneuron fibers wrapped around Purkinje cell bodies (*arrowheads*) is still detected in 4-month-old *Tg* ^*+/−*^ mice. *ML*, molecular layer; *PCL*, PC layer; *GCL*, granule cell layer. *Scale bar* 200 μm (**a**–**b**), 100 μm (**c**, **d**)

**Fig. 8:**
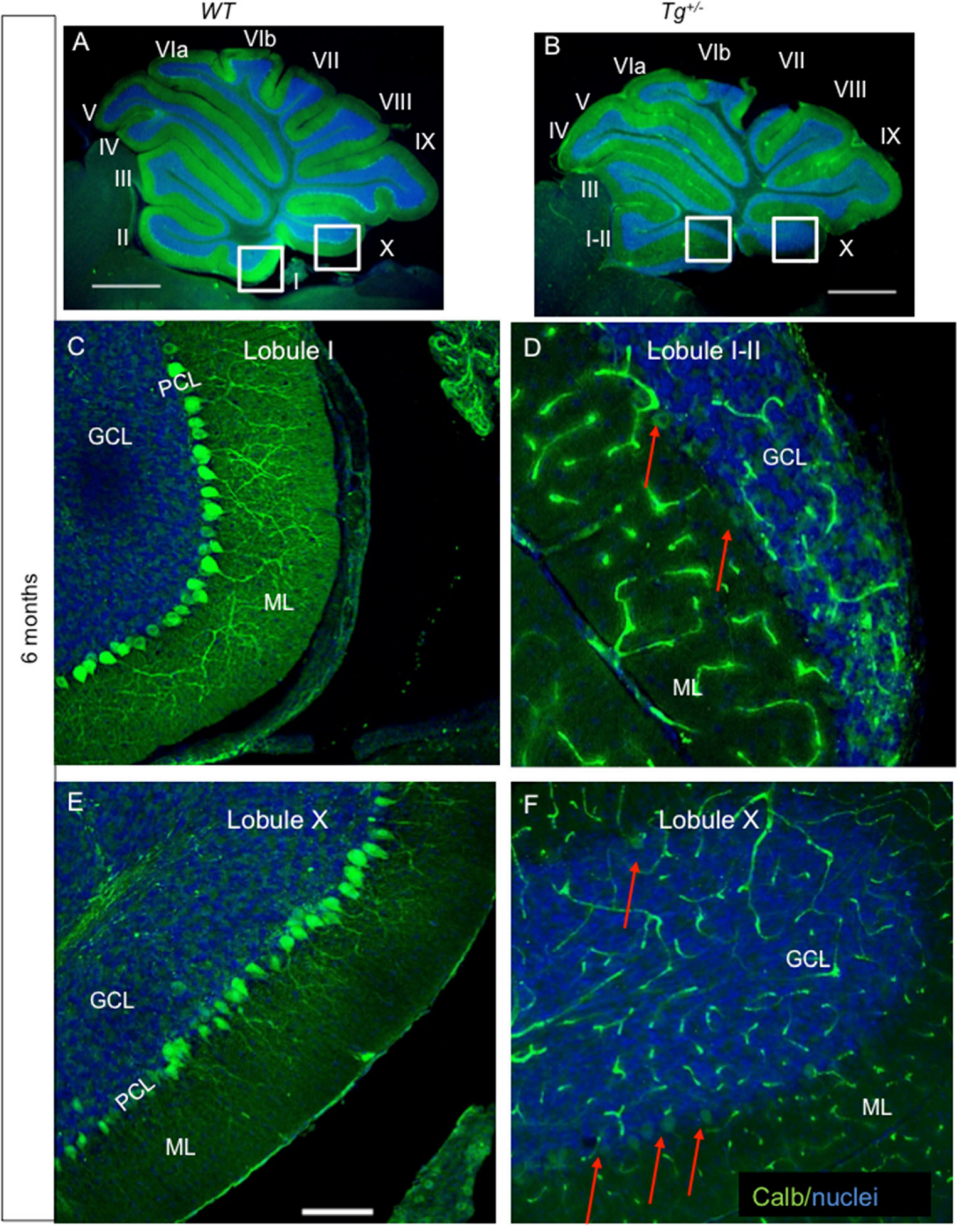
Reduction of Purkinje cell (*PC*) immunogenicity and apparent loss of PC dendrites in the molecular layer of the adult transgenic cerebella. PCs form a monolayer with dense network of dendrites in the ML throughout all the lobules in control cerebellum (**a**). At 6 months, a profound loss of calbindin expression in PCs and PC dendrites in the ML progresses in all lobules of the *Tg* ^*+/−*^ cerebella (**b**) as visualized by lack of staining with anti-calbindin (*green*). A near complete loss of calbindin expression in PCs and PC dendrites (*arrows* in **d**, **f**) is detected in the *Tg* ^*+/−*^ cerebella compared to *WT*, in detail shown in the lobules I–II and X (**c**, **e**). *ML*, molecular layer; *PCL*, PC layer; *GCL*, granule cell layer. *Scale bar* 1000 μm (**a**, **b**); 250 μm (**c**–**f**)

We compared the formation of the Purkinje cell (PC) layer in P16 mice. As in the controls, the PCs were oriented in a monolayer with dendrites projecting into the molecular layer throughout all lobules of the *Tg* ^*+/−*^ cerebellum. However, patches of PCs were missing in the anterior lobe, especially in lobules I–III, and the density of calbindin-labeled PC dendrites appeared to be reduced compared to *WT* littermates (detail of lobule I–II in Fig. 7a, b). With advancing age, PCs progressively lost calbindin immunoreactivity, particularly, in the anterior lobe (Fig. 7c, d). The majority of PCs in lobules I–III lost the expression of calbindin at 4 months of age, although PCs were still present, since basket interneuron fibers (visualized by NF200 staining) were wrapped around PC bodies. At 6 months of age, the deterioration of PCs advanced in all lobules of the transgenic cerebella. The expression of calbindin was significantly diminished in the majority of PCs and their dendrites (Fig. 8a, b), in detail, lobules I–II and X (Fig. 8c–f). Given that there was no profound shrinkage of the molecular layer and Purkinje cell nuclei could still be detected, we presume that PC dendrites are still present but have been reduced or have lost immunopositivity for anti-calbindin. This conclusion is further supported by the presence of scattered patches of preserved PCs, with PC dendrites mostly in lobules V–VIII, and dorsal IX (supplemental file: Fig. S2). A significant attenuation of calbindin expression in PCs of the *Tg* ^*+/−*^ cerebellum may be caused by altered GABA signaling. Changes in calbindin expression may result in an alteration of Ca^2+^ homeostasis with the outcome of altered cerebellar control of motor function as PCs are well known to emit calcium spikes [6].

**Fig. 9:**
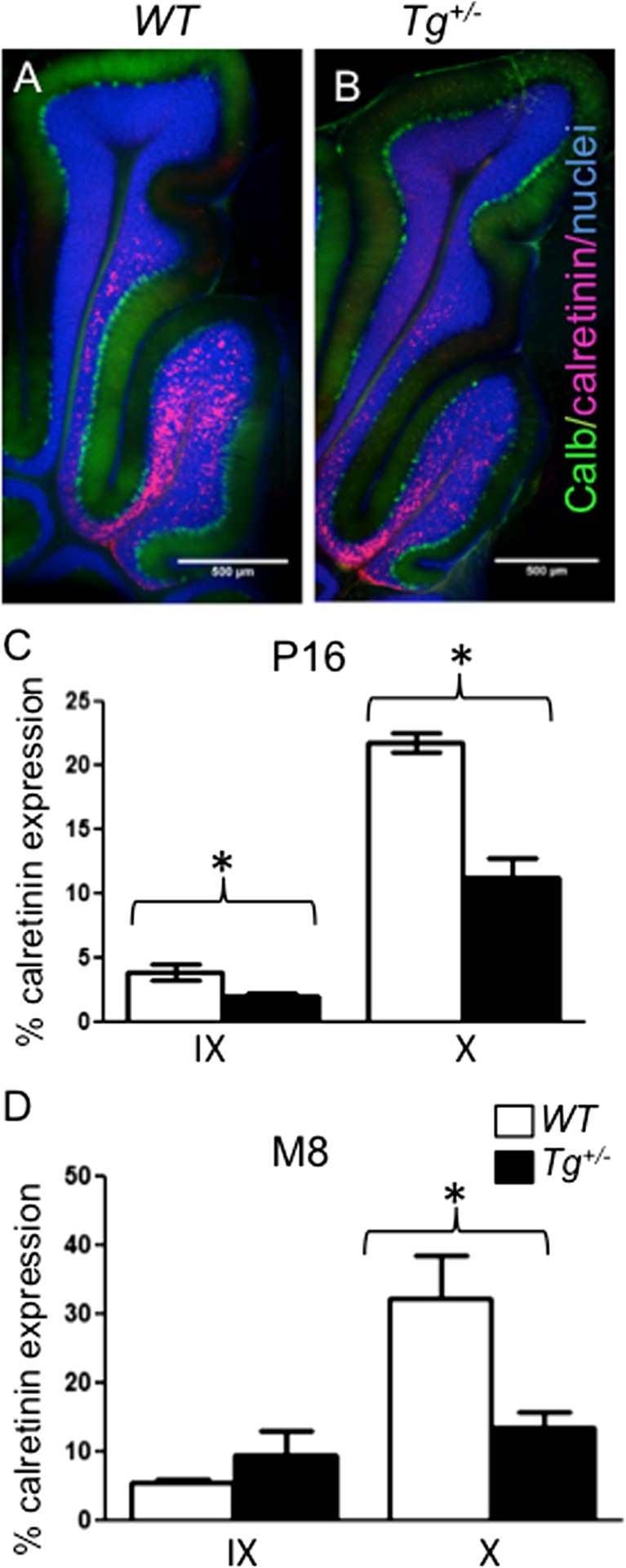
Altered distribution of calretinin-labelled cells in lobules X and IX of the transgenic cerebellum. Calretinin^+^ cells are primarily found in lobules X and half of IX as shown by calretinin staining (*red*) in both *WT* (**a**) and *Tg* ^*+/−*^ (**b**) cerebella. Double staining with anti-Calbindin (*Calb*, *green*) and anti-Calretinin (*red*) and visualization of nuclei with Hoechst staining of 100 μm sections of P16 cerebella. *Scale bar* 500 μm. Quantification of calretinin staining in lobules IX and X of the cerebellum at P16 (**c**) and 8-month-old (**d**) using ImageJ. The values represent an average percentage of calretinin^+^ area/lobule area ± SEM (*n* = 6 *Tg* ^*+/−*^ and 6 *WT*/each age group), *t* test *, *P* < 0.05

Another important calcium-binding protein expressed in the cerebellum is calretinin. Calretinin is expressed predominantly in unipolar brush cells (UBCs) in the posterior lobes (IX, X) of the cerebellum [48, 49]. UBCs receive direct input from the vestibular ganglion and vestibular nuclei [3, 50]. We specifically analyzed calretinin expression in lobules X and IX at P16 and in 8-month-old adult mice. A significant (*P* < 0.05) decrease in calretinin expression was observed in the *Tg* ^*+/−*^ (Fig. 9). The attenuation in calretinin^+^ cells suggests changes in Ca^2+^ homeostasis in cerebellar neurons, which would be expected to affect vestibular information processing in the cerebellum, as UBCs are known to amplify the vestibular input. An alteration of sensory data processing in the cerebellum could affect the behavioral phenotype of *Tg* ^*+/−*^ mice.

**Fig. 10:**
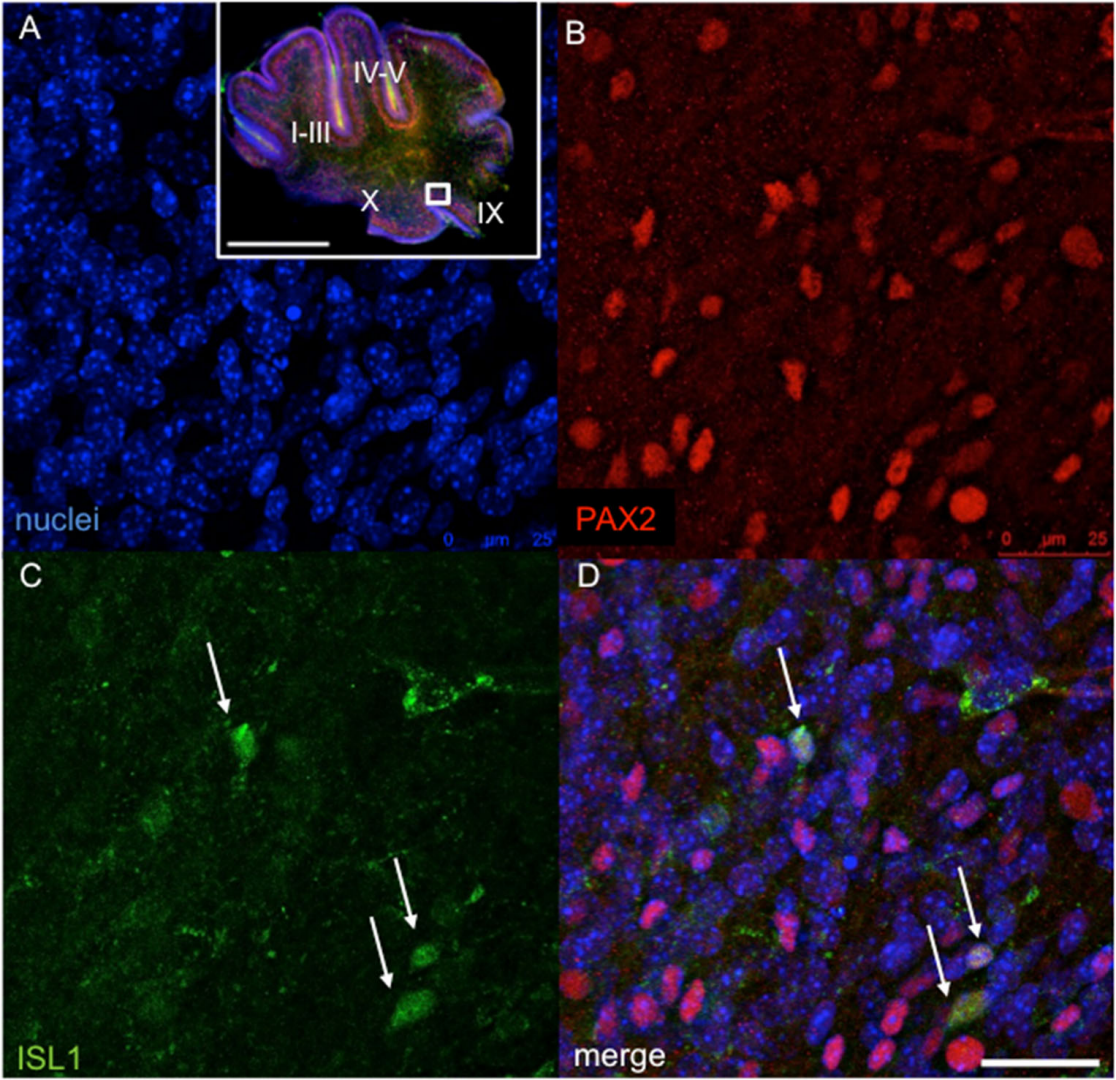
The expression of Isl1 in the transgenic cerebellum at P3. Confocal microscopy of 100 μm sections shows the expression of Isl1 in the transgenic cerebellum (lobule IX) indicated by *white arrows*. Double staining with anti-Pax2 (**b**, *red*) and anti-Isl1 (**c**, *green*) and visualization of nucleus with Hoechst staining (**a**) and overlay of fluorescent channels (**d**). *Scale bar* 500 μm (whole cerebellum), 25 μm (detail **a**–**d**)

**Fig. 11:**
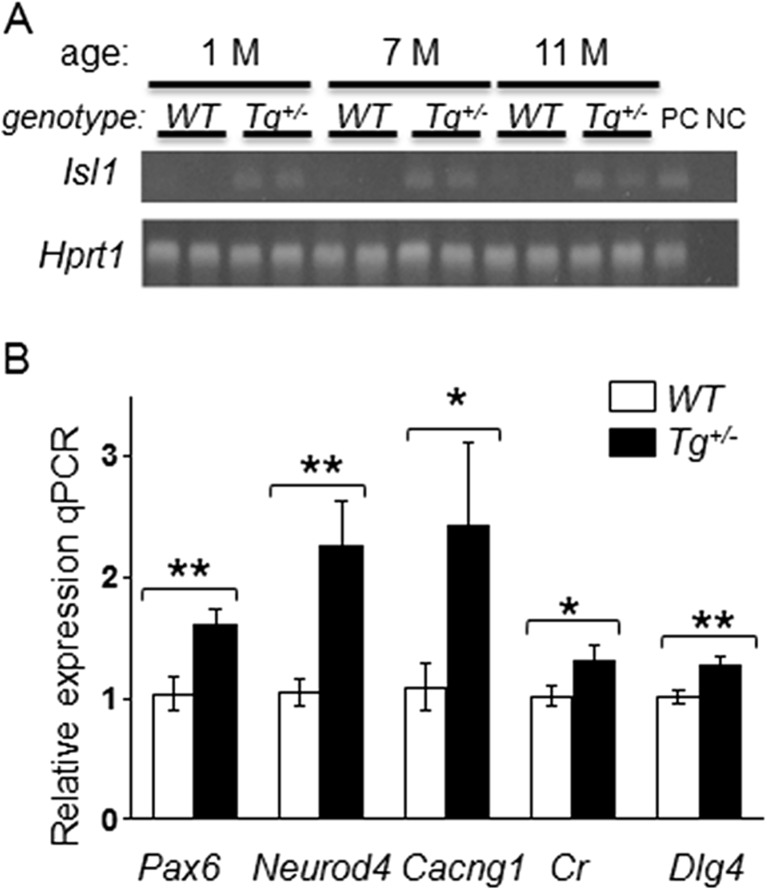
RT-qPCR analysis of gene expression changes induced by the transgenic expression of *Isl1* in the cerebellum. **a** Representative 2 % agarose gel electrophoresis of RT-qPCR products shows the expression of *Isl1* in *Tg* ^*+/−*^ cerebella of 1-, 7-, and 11-month-old mice (two samples/genotype/age). *Hprt1* was used as the reference gene. Lane: *PC*, positive control (hindbrain); *NC*, negative control (H_2_O). **b** The expression of genes was analyzed in *WT* and *Tg* ^*+/−*^ cerebella from 1-month-old mice; the relative expression levels were quantified using −ΔΔCq method. The data represent the expression of mRNA relative to the control cerebella, normalized by the reference gene *Hprt1*. *P < 0.05; **P < 0.01, *t* test. The values are means ± SEM (each experiment in duplicate; *N* = 8/group). *Dlg4*, discs large homolog 4; *Cacng1*, voltage-dependent calcium channel gamma subunit 1; *Cr*, calretinin

Next, we analyzed molecular changes in the *Tg* ^*+/−*^ cerebellum. *Isl1* is expressed in the developing auditory and vestibular neurons [51] but is not expressed in the cerebellum. In order to investigate if the global overexpression of *Isl1* under *Pax2* regulatory sequences led to exogenous expression in the developing cerebellum, we performed immunohistochemistry on sagittal sections of the cerebellar vermis of transgenic mice and their *WT* littermates. *Pax2* identifies the entire population of GABAergic interneurons (basket, stellate, Golgi, and Lugaro cells) in the cerebellar cortex and in the deep cerebellar nuclei [23]. Accordingly, Isl1 protein was detected in the Pax2^+^ cells of the internal granule layer of all lobules at P3 (detail of lobule IX in Fig. 10). This finding was also supported by the detection of *Isl1* mRNA in the *Tg* ^*+/−*^ cerebellum in 1-, 7-, and 11-month-old mice using RT-qPCR (Fig. 11a).

### Gene Expression Profiling in the Cerebellum

In order to further analyze the molecular changes induced by the misexpression of *Isl1*, we analyzed the mRNA expression of selected genes in the cerebellum of 1-month-old mice. We selected a broad spectrum of genes, whose products play a role in the specification and maintenance of different types of neurons (*Atoh1*, *Neurod1*, *Pax6*, *Pax2*, *Shh*, *Ngn2*, *Math3*, *Lhx1*), Ca^2+^ homeostasis (*Cacng1*, calretinin, and parvalbumin), and in neurotransmitter signaling or are structural subunits of the glutamatergic (*Dlg4*, *Slc17a7*, *Grin1*) or GABAergic neurons (*Slc32a1*, *Gphn*). Additionally, we also analyzed the expression of *Isl1* mRNA in the cerebellum and its potential target molecule mRNAs (*Lhx3*, *Neurod4*, *Ngn2*, *Isl2*). Of all the analyzed genes, we detected significant changes in the expression of *Pax6*, *Cacng1*, *Neurod4*, calretinin, and *Dlg4* in the transgenic cerebellum compared to *WT* (Fig. 11b). Thus, the expression profile of transgenic cerebella was significantly altered compared to *WT*.

## Discussion

To explore the gain-of-function role of *Isl1* in the developing cerebellar and vestibular system, we used an overexpression model of *Isl1* under the *Pax2* regulatory sequence. We present data showing that *Isl1* overexpression causes molecular and morphological changes in the cerebellum and vestibular system that may cause hyperactivity, including circling behavior of *Tg* ^*+/−*^ mice. The circling behavior of mutant mice has traditionally been related to vestibular defects (e.g., Bronx-Waltzer mouse [52]) but also to motor control defects in the forebrain, particularly to an imbalance of nigrostriatal function [10]. Below we will first provide the arguments for an ear phenotype being related to circling followed by the correlation of the posterior midbrain and cerebellum with hyperactivity and circling. We suggest that hyperactivity is most likely related to cerebellar malformation, including the progressive loss of calcium binding proteins but not including mild vestibular defects.

### Behavioural Phenotype Associated with Isl1 Transgenic Expression

In tests evaluating behavioral phenotype, *Tg* ^*+/−*^ mice exhibit hyperactivity without balance deficits. In the air-righting test, the mutant mice were indistinguishable from control *WT* mice, suggesting that *Tg* ^*+/−*^ mice do not have a deficit in balance and coordination. Additionally, transgenic mice demonstrated enhanced performance on the accelerating rotarod task than the littermate controls. This was an unexpected finding; however, similar results were reported by other investigators when better performance in the rotarod task was associated with the hyperactivity. For example, heregulin mutants [53], *Pcmt1* ^*−/−*^ [54], or hA53T transgenic mice [55] demonstrate improved performance compared to control *WT* mice in this test. Interestingly, these mice have cerebellar abnormalities and hyperactivity in open-field tests. Hyperactivity without motor abnormalities and superior rotarod performance was also observed in rats with cerebellar neuronal damage (microneuronal hypoplasia) induced by low-dose X-ray radiation [56]. Better rotarod performance is associated with hyperactivity rather than with improved motor functions. These studies, including our data, link cerebellar abnormalities with a hyperactive phenotype.

### The Overexpression of Isl1 Affects the Differentiation of Vestibular End Organs

During early development, vestibular neurons delaminate from the ear and migrate to the vestibular ganglia before projecting back with their dendrites to form the vestibular ganglion located between the ear and brainstem [57]. Consistent with the expression of Isl1 and Pax2 in the sensory neurons of the ear [25, 51], we found a slight acceleration in early fiber development [29], but later innervation of the *Tg* ^*+/−*^ was comparable to *WT* littermates (Fig. 2). In newborn mice, utricle and canal cristae were all labeled at similar intensities. However, the saccule was labeled less (Fig. 2). The saccule is an otolith organ involved in vertical linear movement detection and the sensing of gravity. The saccule shares an embryological origin with the cochlea, arising from the pars inferior of the inner ear [58]. Interestingly, a parallel decline in cochlear and saccular function has been associated with aging in humans [59] and with the shared susceptibility of the saccule and cochlea in pathological processes of Meniere’s disease [60]. Coincidently, the *Tg* ^*+/−*^ mice show cochlear dysfunction as well as reduced size of the saccule that may be associated with the behavioral disorder similar to behavioral disorders observed in individuals with severe inner ear defects [10, 61]. Despite these measurable changes, it seem unlikely that changes in a sensory epithelium dedicated to the perception of vertical alterations in linear acceleration should be responsible for hyperactivity and unilateral rotations described here (Fig. 1). While we cannot rule this out, the data provided below on the cerebellum and midbrain of these transgenic mice correlates better with the behavioral phenotype.

### Morphological and Molecular Changes in the Cerebellum and Midbrain

The cerebellum begins to form at embryonic day 9 (E9) in the mouse and continues through to postnatal development [62]. It is comprised of ten lobules, which are histologically uniform and divided into distinct layers. The analysis of cerebellar morphology of *Tg* ^*+/−*^ showed foliation defects in the anterior lobe, including a partial fusion of lobules I–III, altered layer formation of lobule I fused with the anterior medullary velum, and a defect in the formation of IV/V lobules. In addition, the inferior colliculus was reduced in *Tg* ^*+/−*^. A strikingly similar phenotype was reported in the engrailed1 (*En1*) conditional mutant with *En1* ^*flox*^ allele deleted with the null *En1* ^*Cre*^ knock-in allele [63]. En1 is necessary for the initial formation of the midbrain, and anterior hindbrain and *En1*-null mutants have a complete deletion of this region [64]. *En1* is required for the development of the anterior five cerebellar folia (I–V) and the inferior colliculus. Since *Pax2* and *En1* expression domains overlap and a molecular interaction is needed for the stable differentiation of the isthmus region [27], it is possible that the Isl1 protein of the *Pax2-Isl1* transgene product interacts with En1 signaling to produce a phenotype in the cerebellum and midbrain as in *En1* ^*flox/Cre*^ mutants. This suggestion is supported by similar losses of neurons in *Isl1* and *En1* mutants [15] and other data on Lim protein interactions with En1 proteins [65]. Since our data provide the first in vivo evidence for some direct or indirect interaction of Isl1 and En1 in the cerebellum and midbrain, we suggest that the Isl1 protein partially disables En1 signaling, thus resulting in a similar phenotype (compare Fig. 2 in [63] with our Fig. 4f). Unfortunately, no behavioral details were provided for the *En* mutants [63], and none of the other changes in protein expression we report here have been described in this mutant. Data from functional MRI studies in humans suggests that sensorimotor tasks are processed in lobules IV–V and VIII and that the activation of sensory motor regions is associated with the activation of anterior lobules (I–V) of the cerebellum [66] that also contains its own body representation in humans. We suggest that the cerebellar changes are related to the altered behavior, possibly in combination with the progressive decline of calcium-binding proteins discussed below.

The cerebellum contains five major types of neurons that use either glutamate (granule neurons, UBCs, deep nuclei neurons) or GABA as neurotransmitter (inhibitory interneurons and Purkinje cells). GABAergic neurons originate in the ventricular zone in the roof for the fourth ventricle, and all three glutamatergic types come from the rhombic lip [67]. All three glutamatergic cerebellar neuron types derive from *Pax6* ^*+*^ *, Atoh1* ^*+*^ progenitors [68]. Starting at E13.5, Pax2 is expressed in prospective GABA interneuron precursors in the cerebellar cortex that generate inhibitory interneurons in the cerebellar nuclei, Golgi and Lugaro cells in the granular layer, and basket and stellate cells in the molecular layer [28]. *Isl1* transgenic expression in Pax2^+^ cells might alter cell fate of Pax2^+^ GABAergic neuron population. The aberrations in the cell lineages are further supported by RT-qPCR results showing a significantly altered expression of *Pax6*, *Neurod4*, *Dlg4*, and *calretinin* mRNA in the cerebellum of 1-month-old *Tg* ^*+/−*^. All these genes are associated with glutamatergic neurons suggesting changes in the cell homeostasis of the cerebellum.

The dysfunction in GABA signaling in *Tg* ^*+/−*^ mice is demonstrated by our behavioral studies showing that a subconvulsive dose of picrotoxin normalizes the open-field hyperactive behavior of *Tg* ^*+/−*^ mice. Circling behavior and hyperactivity in mice are also a common presentation of the dysfunction of the striatum [69, 70]. The GABA-mediated striatonigral pathway has been indicated as a major output system from the striatum controlling circling activity [71]. Although our analysis of Isl1 expression in the striatum at E14.5 did not show any differences, we cannot exclude an attenuation in the input from the vestibular system that may cause a change in the striatum and/or striatonigral pathway of *Tg* ^*+/−*^ resulting in hyperactivity and circling.

Purkinje cells belong to GABAergic neuronal subtypes; however, their progenitors do not express *Pax2* [72]. Purkinje cells play a key role in connectivity forming a cortico-nucleo-olivary loop important for motor behavior [6]. Proper connectivity is critical for motor coordination, and the degeneration of the cerebellar circuits is associated with several neurological degenerative diseases. With increasing age, we detected reduced Purkinje cell calbindin expression in the transgenic cerebellum. This may indicate that Isl1 also affects the maintenance of Purkinje cells and their calcium homeostasis.

Another important calcium binding protein is calretinin, which is expressed in UBC, Lugaro-like, granular, Purkinje, and astrocyte cells of the cerebellum [49]. We specifically analyzed calretinin expression in the vermis of lobule X and the ventral portion of lobule IX, where UBCs are particularly concentrated [50]. UBCs are a distinct type of glutamatergic interneurons in the cerebellar cortex and cochlear nucleus. It is thought that they serve as amplifiers of vestibular signals through a powerful feed-forward link due to the transfer of a signal from a single mossy fiber to a number of neighboring granule cells [3, 73]. A significant decrease in calretinin expression was observed in the lobule X and IX of *Tg* ^*+/−*^ compared to *WT* littermates at P16 and in adults (Fig. 9). This could be explained by UBC cell death, possibly as a result of altered innervation from the saccule [3]. Conversely, the reduction in the number of calretinin^+^ cells may reflect the downregulation of calretinin expression as a response to a decrease in sensory input. The effect could also be a consequence of aberrations in the cell lineages of the Pax2^+^ precursors. The impairment of Ca^2+^ homeostasis in Purkinje cells as well as the reduction of calretinin-mediated Ca^2+^ buffering would predict modifications in intracellular calcium concentration resulting in altered information processing and thus motor alterations such as hyperactivity.

## Conclusion

Based on our behavioral study, the transgenic expression of Isl1 specifically affects GABA signaling. We found that Isl1 overexpression in the developing vestibular ear results in a smaller saccule with a significantly reduced number of hair cells and innervation. We observed both morphological and molecular changes in the cerebellum, especially at the vestibule-cerebellum and the anterior lobe, which may be associated with altered functions and abnormal behavior of the *Tg* ^*+/−*^ mice. Additionally, in the *Tg* ^*+/−*^ midbrain, the inferior colliculus was severely reduced. Taken together, the development of the cerebellum, midbrain, and the vestibular end organs is altered by the transgenic expression of Isl1. It is intriguing to consider whether an alternation of transcription regulation in the development of the vestibular system may contribute to psychiatric and motor disorders that show correlation with the shrinking of the anterior lobe of the cerebellum [74]. A most interesting correlation exists between our hyperactive mice and the age-related shrinking of the cerebellum [75] and altered GABA signaling [76, 77] in people with attention deficit hyperactivity disorder (ADHD). It remains to be seen if our Isl1 transgenic mice can serve as a model for ADHD. We are currently evaluating standard treatment of ADHD such as Ritalin for its effect on our transgenic mice [78].

## Electronic Supplementary Material

Below is the link to the electronic supplementary material.

Table S1 (DOCX 19 kb)

Fig. S1 Dorsal view of the adult brain. The inferior colliculus (*IC*) was significantly reduced in the transgenic brain compared to *WT*. The superior colliculus (*SC*) and IC are outlined by *blue* and *red dashed lines*, respectively. *Cb*, cerebellum; *V*, vermis; *H*, hemisphere (GIF 341 kb)

High resolution image (TIFF 2927 kb)

Fig. S2 Reduction of Purkinje cells (*PC*) immunogenicity and an apparent loss of PC dendrites in the molecular layer of adult transgenic cerebella. A profound reduction of calbindin expression in PCs and PC dendrites in the molecular layer is detected in all lobules of the *Tg* ^*+/−*^ cerebella (**b**) as visualized by the lack of staining with anti-calbindin (*green*) compared to *WT* (**a**). However, some scattered patches of PCs with dendrites are still preserved in *Tg* ^*+/−*^ as shown in lobule VIII of (**d**) similar to *WT* (**c**) at 6-month-old cerebella. *ML*, molecular layer; *PCL*, PC layer; *GCL*, granule cell layer. *Scale bar* 1000 μm (**a**, **b**); 250 μm (**c**, **d**) (GIF 744 kb)

High resolution image (TIFF 2927 kb)

ESM 1 (MPG 9824 kb)

ESM 2 (MPG 14240 kb)

ESM 3 (MPG 6976 kb)

ESM 4 (MPG 9696 kb)
