# Supplementary material for: Pax2-Islet1 Transgenic Mice Are Hyperactive and Have Altered Cerebellar Foliation
Source: Mol Neurobiol. 2016 Feb 3;54(2):1352–68. doi: 10.1007/s12035-016-9716-6 (PMC5310572; doi:10.1007/s12035-016-9716-6)
Supplement: Supplementary file 1 — (DOCX 19 kb) [file 12035_2016_9716_MOESM5_ESM.docx]

Table S1. Primer sequences

| Hprt1* | Forward | 5'-GCTTGCTGGTGAAAAGGACCTCTCGAAG-3' |
| --- | --- | --- |
|  | Reverse | 5'-CCCTGAAGTACTCATTATAGTCAAGGGCAT-3' |
| Isl1* | Forward | 5'-GCAACCCAACGACAAAACTAA-3' |
|  | Reverse | 5'-CCATCATGTCTCTCCGGACT-3' |
| Atoh1 | Forward | 5'-GAGTGGGCTGAGGTAAAAGAGT-3' |
|  | Reverse | 5'-GGTCGGTGCTATCCAGGAG-3' |
| Neurod1 | Forward | 5'-ATGACCAAATCATACAGCGAGAG-3' |
|  | Reverse | 5'-TCTGCCTCGTGTTCCTCGT-3' |
| Shh | Forward | 5'-AAAGCTGACCCCTTTAGCCTA-3' |
|  | Reverse | 5'-TTCGGAGTTTCTTGTGATCTTCC-3' |
| Pax6 | Forward | 5'-TACCAGTGTCTACCAGCCAAT-3' |
|  | Reverse | 5'-TGCACGAGTATGAGGAGGTCT-3' |
| Lhx3* | Forward | 5'-CAGACCCAGGGGAAGTTCAG-3' |
|  | Reverse | 5'-CACACATCGGGATCTCTCGG-3' |
| Neurod4 | Forward | 5'-AGCTGGTCAACACACAATCCT-3' |
|  | Reverse | 5'-GTTCCGAGCATTCCATAAGAGC-3' |
| Neurog2 | Forward | 5'-AACTCCACGTCCCCATACAG-3' |
|  | Reverse | 5'-GAGGCGCATAACGATGCTTCT-3' |
| Isl2* | Forward | 5'-GCGGGGCAGGAGTTAGTTAG-3' |
|  | Reverse | 5'-CCGGGCTTCTTCTTGGAATG-3' |
| Calb2* | Forward | 5'-TGATGCTGACGGAAATGGGT-3' |
|  | Reverse | 5'-CCCTTCCTTGCCTTCTCCAG-3' |
| Pvalb | Forward | 5'-ATCAAGAAGGCGATAGGAGCC-3' |
|  | Reverse | 5'-GGCCAGAAGCGTCTTTGTT-3' |
| Cacng1 | Forward | 5'-AAAACAGCGAAGGTTCGTGTG-3' |
|  | Reverse | 5'-CTGCCTCGCACGTTTCATTG-3' |
| Pax2 | Forward | 5'-AAGCCCGGAGTGATTGGTG-3' |
|  | Reverse | 5'-CAGGCGAACATAGTCGGGTT-3' |
| Lhx1 | Forward | 5'-CCCATCCTGGACCGTTTCC-3' |
|  | Reverse | 5'-CGCTTGGAGAGATGCCCTG-3' |
| Slc32a1 | Forward | 5'-ACCTCCGTGTCCAACAAGTC-3' |
|  | Reverse | 5'-CAAAGTCGAGATCGTCGCAGT-3' |
| Gphn | Forward | 5'-CAACCACGACCATCAAATCCG-3' |
|  | Reverse | 5'-CCAACAAAGAAGGATCTTGGACA-3' |
| Grin1 | Forward | 5'-AGAGCCCGACCCTAAAAAGAA-3' |
|  | Reverse | 5'-CCCTCCTCCCTCTCAATAGC-3' |
| Slc17a7 | Forward | 5'-GGTGGAGGGGGTCACATAC-3' |
|  | Reverse | 5'-AGATCCCGAAGCTGCCATAGA-3' |
| Dlg4 | Forward | 5'-TGAGATCAGTCATAGCAGCTACT-3' |
|  | Reverse | 5'-CTTCCTCCCCTAGCAGGTCC-3' |

*Primers designed using <http://www.ncbi.nlm.nih.gov/tools/primer-blast/>; the rest of primers from primer database http://pga.mgh.harvard.edu/primerbank
