# Supplementary figures and images for: Pax2-Islet1 Transgenic Mice Are Hyperactive and Have Altered Cerebellar Foliation
Source: Mol Neurobiol. 2016 Feb 3;54(2):1352–68. doi: 10.1007/s12035-016-9716-6 (PMC5310572; doi:10.1007/s12035-016-9716-6)

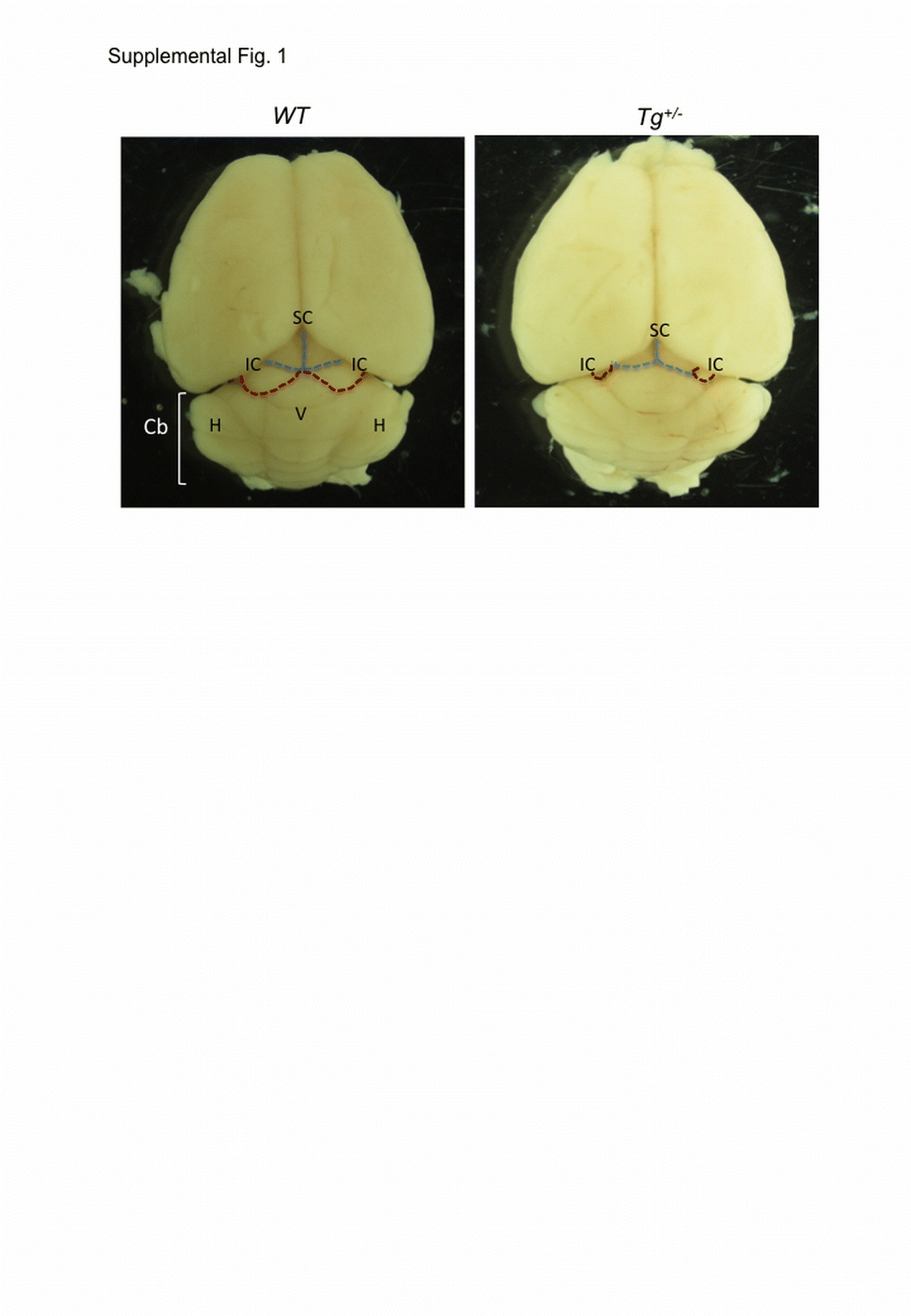

Supplement: Supplementary file 2 — Dorsal view of the adult brain. The inferior colliculus (IC) was significantly reduced in the transgenic brain compared to WT. The superior colliculus (SC) and IC are outlined by blue and red dashed lines, respectively. Cb, cerebellum; V, vermis; H, hemisphere (GIF 341 kb) [file 12035_2016_9716_Fig12_ESM.gif]

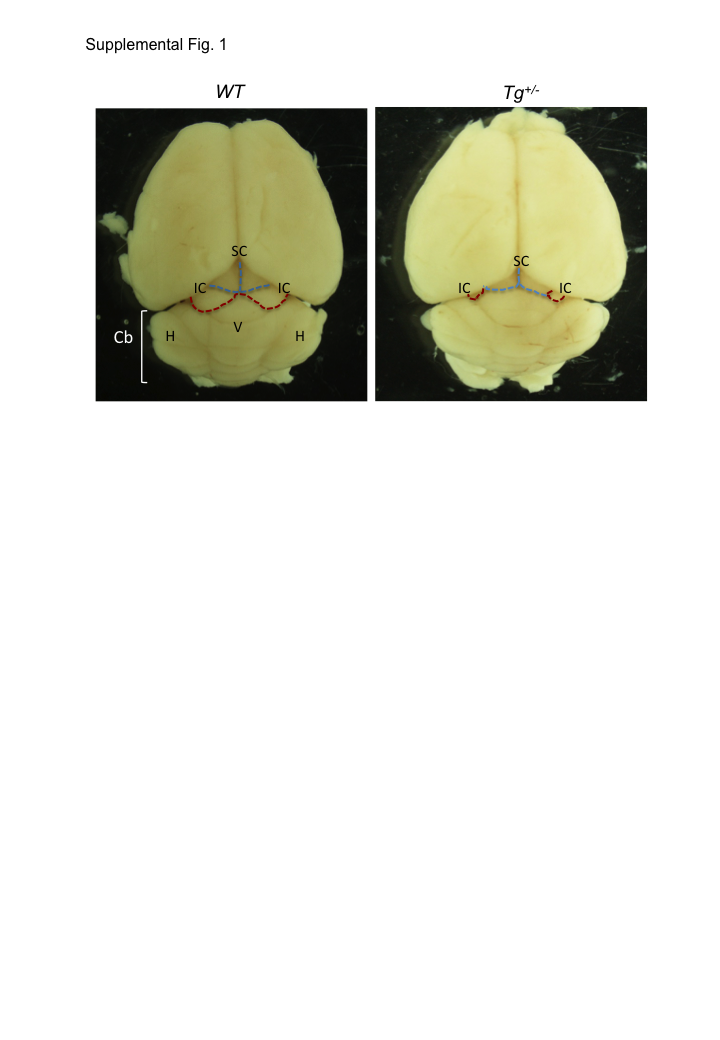

Supplement: Supplementary file 3 — High resolution image (TIFF 2927 kb) [file 12035_2016_9716_MOESM6_ESM.tiff]

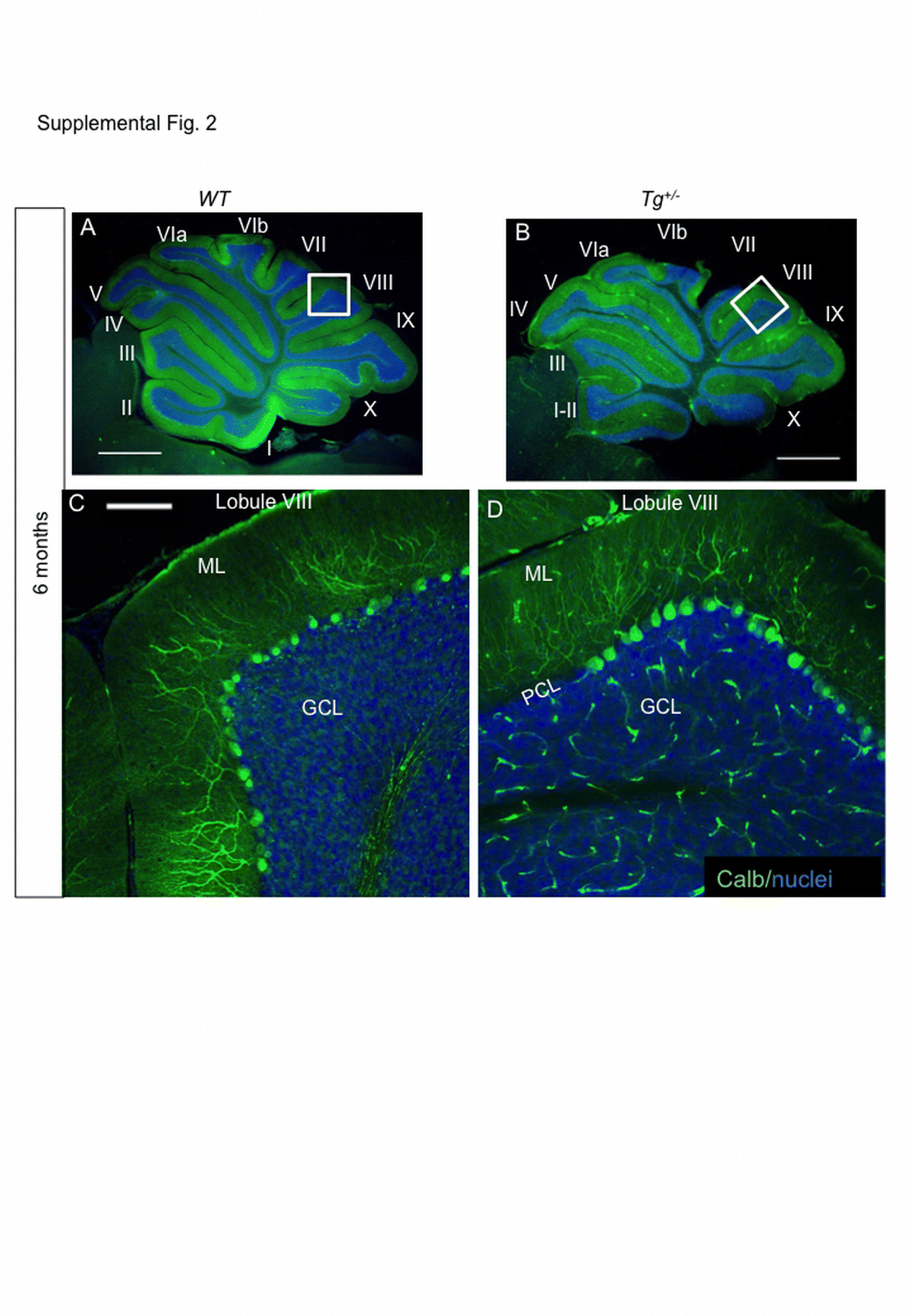

Supplement: Supplementary file 4 — Reduction of Purkinje cells (PC) immunogenicity and an apparent loss of PC dendrites in the molecular layer of adult transgenic cerebella. A profound reduction of calbindin expression in PCs and PC dendrites in the molecular layer is detected in all lobules of the Tg +/− cerebella (b) as visualized by the lack of staining with anti-calbindin (green) compared to WT (a). However, some scattered patches of PCs with dendrites are still preserved in Tg +/− as shown in lobule VIII of (d) similar to WT (c) at 6-month-old cerebella. ML, molecular layer; PCL, PC layer; GCL, granule cell layer. Scale bar 1000 μm (a, b); 250 μm (c, d) (GIF 744 kb) [file 12035_2016_9716_Fig13_ESM.gif]

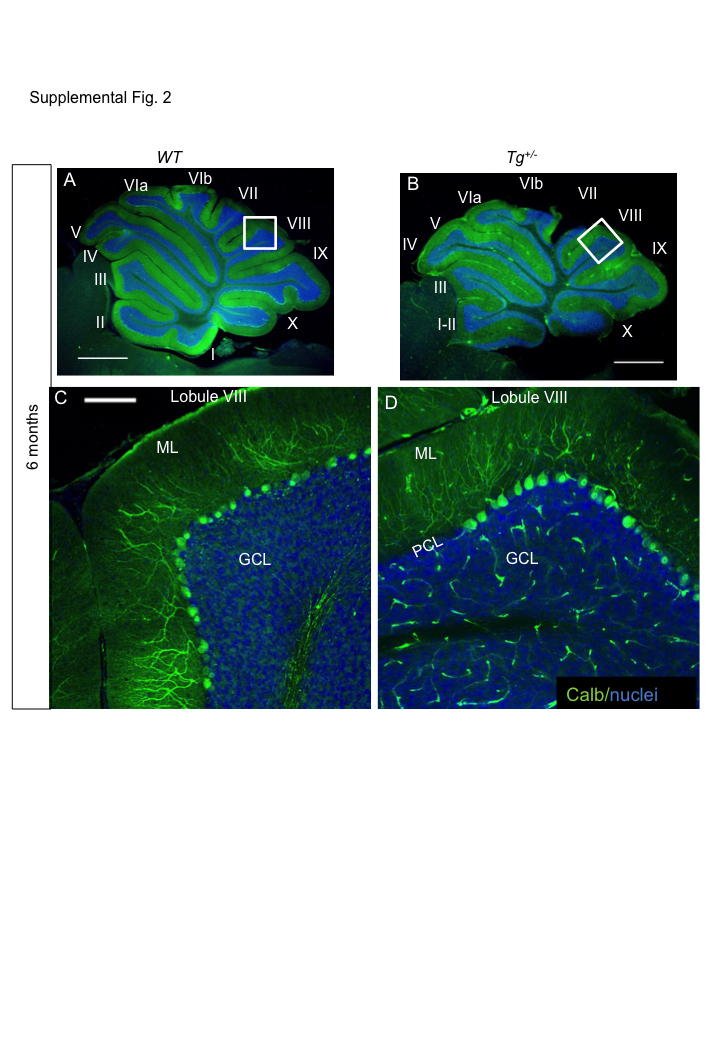

Supplement: Supplementary file 5 — High resolution image (TIFF 2927 kb) [file 12035_2016_9716_MOESM7_ESM.tiff]
